# Disease burden and economic impact of diagnosed non‐alcoholic steatohepatitis in five European countries in 2018: A cost‐of‐illness analysis

**DOI:** 10.1111/liv.14825

**Published:** 2021-03-18

**Authors:** Jörn M. Schattenberg, Jeffrey V. Lazarus, Philip N. Newsome, Lawrence Serfaty, Alessio Aghemo, Salvador Augustin, Emmanuel Tsochatzis, Victor de Ledinghen, Elisabetta Bugianesi, Manuel Romero‐Gomez, Heike Bantel, Stephen D. Ryder, Jerome Boursier, Vincent Leroy, Javier Crespo, Laurent Castera, Lefteris Floros, Vincenzo Atella, Jorge Mestre‐Ferrandiz, Rachel Elliott, Achim Kautz, Alice Morgan, Sally L. Sansom, Sharad Vasudevan, Lynne Pezzullo, Aldo Trylesinski, Sandrine Cure, Victoria Higgins, Vlad Ratziu

**Affiliations:** ^1^ Metabolic Liver Research Center, I. Department of Medicine University Medical Center Mainz Germany; ^2^ Barcelona Institute for Global Health (ISGlobal) Hospital Clínic, University of Barcelona Barcelona Spain; ^3^ National Institute for Health Research Biomedical Research Centre University Hospitals Birmingham NHS Foundation Trust and the University of Birmingham Birmingham UK; ^4^ Centre for Liver and Gastrointestinal Research, Institute of Immunology and Immunotherapy University of Birmingham Birmingham UK; ^5^ Liver Unit University Hospitals Birmingham NHS Foundation Trust Birmingham UK; ^6^ Hôpitaux Universitaires de Strasbourg Strasbourg France; ^7^ Humanitas University and Humanitas Clinical and Research Center – IRCCS via Alessandro Manzoni 56, I20089 Rozzano Milan Italy; ^8^ Hospital Universitari Vall d'Hebron – Institut de Recerca Barcelona Spain; ^9^ UCL Institute for Liver and Digestive Health Royal Free Hospital London UK; ^10^ Centre Hospitalier Universitaire Bordeaux Bordeaux France; ^11^ Department of Medical Sciences University of Torino Torino Italy; ^12^ Virgen del Rocío University Hospital Sevilla Spain; ^13^ Medizinische Hochschule Hannover Hannover Germany; ^14^ National Institute for Health Research Nottingham Biomedical Research Centre at Nottingham University Hospitals and the University of Nottingham Nottingham UK; ^15^ Angers University Hospital Angers France; ^16^ Centre Hospitalier Universitaire de Grenoble Grenoble France; ^17^ Hospital Universitario Marqués de Valdecilla Santander Spain; ^18^ Department of Hepatology, Hôpital Beaujon Université Paris‐7 Paris France; ^19^ PHMR Limited London UK; ^20^ University Rome Tor Vergata Rome Italy; ^21^ Independent Economics Consultant Madrid Spain; ^22^ University of Manchester Manchester UK; ^23^ Kautz^5^ Köln Germany; ^24^ Deloitte Canberra Australia; ^25^ Deloitte Victoria Australia; ^26^ Intercept Pharmaceuticals London UK; ^27^ Adelphi Real World Cheshire UK; ^28^ Hôpital de la Pitié‐Salpêtrière Paris France

**Keywords:** burden of disease, cost‐of‐illness analysis, economic impact, healthcare resource utilisation, non‐alcoholic steatohepatitis (NASH)

## Abstract

**Background and aims:**

Non‐alcoholic steatohepatitis (NASH) is a chronic disease that can progress to end‐stage liver disease (ESLD). A large proportion of early‐stage NASH patients remain undiagnosed compared to those with advanced fibrosis, who are more likely to receive disease management interventions. This study estimated the disease burden and economic impact of diagnosed NASH in the adult population of France, Germany, Italy, Spain and the United Kingdom in 2018.

**Methods:**

The socioeconomic burden of diagnosed NASH was estimated using cost‐of‐illness methodology applying a prevalence approach to estimate the number of adults with NASH and the attributable economic and wellbeing costs. Given undiagnosed patients do not incur costs in the study, the probability of diagnosis is central to cost estimation. The analysis was based on a literature review, databases and consultation with clinical experts, economists and patient groups.

**Results:**

The proportion of adult NASH patients with a diagnosis ranged from 11.9% to 12.7% across countries, which increased to 38.8%‐39.1% for advanced fibrosis (F3‐F4 compensated cirrhosis). Total economic costs were €8548‐19 546M. Of these, health system costs were €619‐1292M. Total wellbeing costs were €41 536‐90 379M. The majority of the undiagnosed population (87.3%‐88.2% of total prevalence) was found to have early‐stage NASH, which, left untreated, may progress to more resource consuming ESLD over time.

**Conclusions:**

This study found that the majority of economic and wellbeing costs of NASH are experienced in late disease stages. Earlier diagnosis and care of NASH patients could reduce future healthcare costs.

**Abbreviations**
AASLD: American Association for the Study of Liver Disease
AFP: alpha‐fetoprotein
CC: compensated cirrhosis
DALY: disability‐adjusted life year
DCC: decompensated cirrhosis
DSP: Adelphi NASH Disease Specific Programme^™^
EASL: European Association for the Study of the Liver
ESLD: end‐stage liver disease (DCC, HCC and liver transplant)
F0: fibrosis stage 0
F0‐DCC: fibrosis stages which include measurement of the amount of liver fibrosis
F1: fibrosis stage 1
F2: fibrosis stage 2
F3: fibrosis stage 3
F4 CC: fibrosis stage 4 compensated cirrhosis
FEKI: Freiburg Ethics Commission International
HCC: hepatocellular carcinoma
IHME: Institute for Health Metrics and Evaluation
MEPS: Medical Expenditure Panel Survey
NAFLD: non‐alcoholic fatty liver disease
NASH: non‐alcoholic steatohepatitis
NICE: National Institute for Health and Care Excellence
UK: United Kingdom
US: United States
VSLY: value of a statistical life year
WHO: World Health Organization
WPAI: Work Productivity and Activity Impairment
YLD: years of healthy life lost owing to disability
YLL: years of life lost owing to premature death

**Key points**

- This study estimated the disease burden and economic impact of diagnosed non‐alcoholic steatohepatitis (NASH) in the adult population of France, Germany, Italy, Spain and the United Kingdom in 2018.
- As the majority of economic and wellbeing costs are experienced in late disease stages, earlier diagnosis and care of patients with NASH could reduce future healthcare and wellbeing costs.

## 1 INTRODUCTION

Non‐alcoholic fatty liver disease (NAFLD) is a chronic liver disease characterised by excessive fat deposition in the liver in the absence of competing liver disease aetiologies, such as alcohol‐related liver disease or chronic viral hepatitis.^1^, ^2^ The epidemic of obesity and type 2 diabetes in Europe and the United States (US) has led to an increasing prevalence of NAFLD, which is now one of the most frequent causes of chronic liver disease and one of the leading causes of cirrhosis and liver transplantation for end‐stage liver disease (ESLD).^3^ Non‐alcoholic steatohepatitis (NASH)—the progressive, inflammatory form of NAFLD—is widely considered to be the hepatic manifestation of metabolic syndrome.^2^ NASH is defined by changes observed on liver histology and includes the presence of ≥5% hepatic steatosis and inflammation with hepatocyte injury (eg ballooning), with varying degrees of fibrosis. Given that it is not feasible to conduct liver biopsies in studies of the general population (owing to practical, cost and ethical considerations), there is no direct assessment of the prevalence or incidence of NASH in the epidemiological literature. Patients with advanced fibrosis from NASH are at greater risk of progressing to ESLD and, thus, this population potentially exhibits the greatest disease burden and economic costs.^2^ The costs associated with NASH are likely to increase overtime in parallel with increasing disease prevalence.^2^, ^3^, ^4^, ^5^, ^6^, ^7^, ^8^ These costs can be classified as economic costs through their effects on healthcare, productivity and carers. Furthermore, administrative inefficiencies are associated with raising taxation revenue (which would otherwise be collected from NASH patients, their carers and employers) in order to fund government expenditures including healthcare and welfare benefits. These inefficiencies may be monetised and are referred to as deadweight losses. NASH also negatively affects wellbeing and adds to premature morbidity and mortality.^8^ To quantify these costs, a comprehensive exploration of economic and wellbeing‐associated costs is warranted.

This study estimated the disease burden and economic impact of adults diagnosed with NASH in France, Germany, Italy, Spain and the United Kingdom (UK) in 2018. This type of cost‐of‐illness analysis may support measures to address an increasingly prevalent, yet mostly asymptomatic disease.^9^

## 2 MATERIALS AND METHODS

**FIGURE 1:**
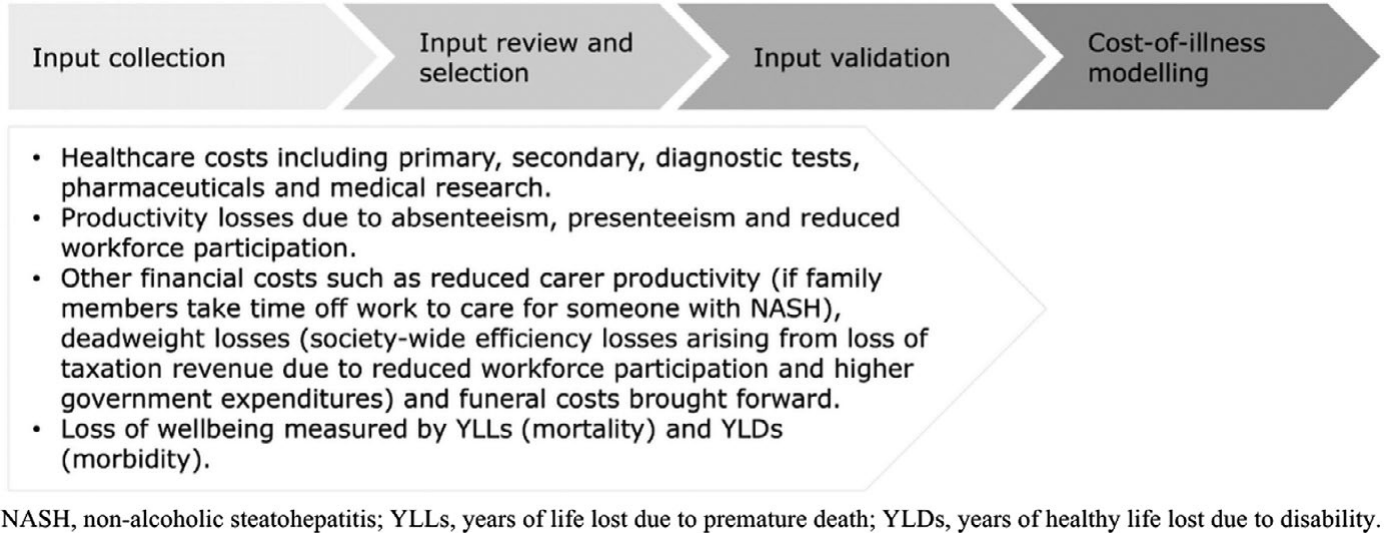
High‐level materials and method flowchart (uploaded as File S1)

The socioeconomic burden of diagnosed NASH in the five European countries was estimated from a societal perspective using a cost‐of‐illness methodology applying a diagnosed prevalence approach.^10^ This method has been described in detail previously and is briefly summarised in Figure 1. At a high‐level, this involved estimating the number of people with diagnosed NASH in a base period (2018) and the economic and wellbeing costs attributable to this condition during the base year. Only patients diagnosed with NASH incurred costs in the model. Costs attributable to comorbidities associated with NASH were excluded.

A review of the scientific literature was conducted to obtain inputs for modelling, supplemented with information obtained from existing databases and through consultation with 31 expert stakeholders including 18 clinical experts, 9 health economists and 4 patient group representatives via 5 separate in‐person workshops and follow‐up consultations. Table S1 outlines the details of experts on each panel for each country.

The data available to estimate the disease burden of NASH were limited and imperfect, requiring a considered methodological approach in the selection of inputs for the modelling. As such, the selection of inputs for this study followed a systematic hierarchical approach considering three factors: quality, generalisability and internal consistency. NASH and country‐specific inputs were used where available. Otherwise, each data point was considered in terms of its associated quality, generalisability and internal consistency.

The inputs selected were validated with the aforementioned expert panel in each of the five European countries via a series of consultations leveraging principles of expert elicitation, as previously described.^11^, ^12^ Experts were tasked with validating estimates of the proportion of patients diagnosed with NASH at any stage following liver biopsy and/or non‐invasive diagnosis in their countries based on clinical practice and their expertise. These estimates were cross checked against literature where data were available. Furthermore, inputs relating to the probability of diagnosis and health system utilisation underwent stepwise validation given the lack of available peer‐reviewed literature and published data on these topics. Where available, national estimated figures of hepatocellular carcinomas and liver transplantations formed the basis of estimates. The outcome of this provided consensus among the expert groups regarding the clinical practice (diagnosis and management) of NASH patients within each country.

Given that inputs were validated with experts within each country, we did not require all inputs to be consistently defined across countries. For example, the consensus led to some variation in how NASH was diagnosed in each country. NASH patients in the UK received a selection of diagnostic tests including liver function test, ultrasound and elastography and alpha‐fetoprotein (AFP) in the context of hepatocellular carcinoma (HCC) screening (Table S12). In Germany, NASH patients also received a fibrosis score, and in selected cases computed tomography and magnetic resonance imaging as part of the diagnostic process including workup for suspected HCC. The limitations of non‐standardised diagnostic approaches across European Union countries are described more fully in the discussion.

### 2.1 Epidemiology of NASH

NASH prevalence estimates (lower and higher scenarios) by age, gender and disease stage, and other epidemiological parameters including incidence, attributable liver transplants and attributable mortality were obtained from peer‐reviewed literature and other published data.^2^, ^3^, ^4^, ^5^, ^6^, ^7^, ^18^ Nine disease stages were included in the analysis—fibrosis stage 0 (F0), fibrosis stage 1 (F1), fibrosis stage 2 (F2), fibrosis stage 3 (F3), fibrosis stage 4 compensated cirrhosis (F4 CC), decompensated cirrhosis (DCC), HCC, liver transplant and death (including both liver‐ and cardiovascular‐related death).

An upper scenario estimate of the total prevalence rate of NASH in the adult population of the five European countries was derived from Estes et al^5^. The overall population prevalence of NASH in the five European countries in 2016 was assumed to be representative of the respective prevalence rates in 2018. Lower scenario sensitivity analysis was conducted by applying an alternate published modelled prevalence rate to the disease stage, age and gender distribution of NASH.^2^ Age distribution was based on a US modelling study by Younossi et al for all disease stages, scaled to the overall prevalence.^6^ Sex distribution was based on the NAFLD population, as reported in Lazo et al, to all disease stages.^13^ These derived prevalence rates were applied to population estimates by age and gender from country‐specific sources.^14^, ^15^ The incidence of NASH by disease stage was used to derive the newly diagnosed and monitored populations, assuming a proportionate diagnosis rate.^16^

Liver transplants attributable to NASH were applied to the age and sex distribution of NASH prevalence.^18^ People aged 70 and over were assumed to be precluded from receiving a liver transplant,^17^ an assumption that was validated with experts in each study country. Liver‐related and excess cardiovascular disease mortality was based on Estes et al.^5^ Excess cardiovascular disease mortality was estimated to comprise 60% of total attributable mortality according to the published data.^5^

#### 2.1.1 Diagnosis of NASH

The diagnosis rates of NASH were determined based on consultation with clinical experts, highlighting that NASH diagnoses are primarily made incidentally during routine clinical investigations for incident liver function test abnormalities or steatosis on ultrasound. Expert opinion was elicited via a standardised process that was validated through other sources, where available. However, wide variation and uncertainty in diagnosis rates among the five countries persisted, hence the need to explore prevalence scenarios through sensitivity testing. It was suggested that a large proportion of the prevalent NASH population were undiagnosed prior to developing advanced fibrosis or cirrhosis. This is important as disease‐specific intervention can only be offered after a NASH diagnosis has been established. In the absence of a NASH diagnosis, no disease‐modifying interventions will be offered (refer to File S1).^11^, ^12^, ^17^, ^19^

The probability of diagnosis at each disease stage was initially derived from a UK economic evaluation of an alternative diagnostic pathway, which reported the probability of detection from a UK prospective cross‐sectional feasibility study.^17^, ^19^ The estimates were presented and validated with a panel of clinical experts in five European countries via a series of consultations leveraging principles of expert elicitation and amended to reflect local clinical practice in each country. This involved presenting the panel with the estimated number of prevalent and diagnosed patients using the aforementioned country‐specific sources. The panel drew from their knowledge of the number of diagnosed patients within their collective care and the number and size of specialist centres in their country to provide feedback on the estimates. Revised estimates were shared with the panel following their feedback for validation. This modified Delphi technique was repeated until consensus was achieved within each country. These derived estimates are applied throughout the results section (derived probability of diagnosis scenario). Sensitivity analysis on the probability of diagnosis was conducted via lower and higher scenarios where these scenarios represent the application of the lowest/highest derived probability of diagnosis scenario across all included countries. This means that in the lower scenario, the derived probability of diagnosis scenario for Spain was applied to all countries, while, in the higher scenario, the derived probability of diagnosis scenario for Germany was applied to all countries. File S1 outlines the results of a lower and higher probability of diagnosis scenario (Tables S2‐S11) applied to the lower and higher prevalence scenarios for each country. Variation in the revised probabilities of diagnosis may be as a result of a combination of factors including country‐specific differences in clinical practice and perceptions of the number of ‘silent’ NASH patients in each country.

### 2.2 Economic costs of diagnosed NASH

#### 2.2.1 Health system costs of diagnosed NASH

Health system costs include primary and secondary healthcare, diagnostic tests, pharmaceuticals and medical research.^17^, ^20^, ^21^, ^22^, ^23^, ^24^, ^25^, ^26^, ^27^, ^28^, ^29^, ^30^, ^31^, ^32^, ^33^, ^34^, ^35^, ^36^, ^37^, ^38^, ^39^, ^40^, ^41^, ^42^, ^43^, ^44^, ^45^, ^46^, ^47^, ^48^, ^49^, ^50^, ^51^, ^52^, ^53^, ^54^ Health system costs were estimated for patients who were ‘newly diagnosed’ and ‘monitored’ within each disease stage. Newly diagnosed refers to patients who, in 2018, are in their first year of care following diagnosis. Monitored refers to patients who, in 2018, are in their second or subsequent years of care, following a previous diagnosis. Health system costs by disease stage and type were calculated by applying the average, per‐person health system cost for that disease stage to the respective diagnosed population. Per‐person costs are slightly higher in the lower prevalence scenario as fixed costs are distributed over a smaller population.

Total health system costs for patients in the liver transplant disease stage in the UK, and DCC, HCC and liver transplant disease stages in France, Germany, Italy and Spain were obtained from studies estimating the costs of these disease stages (ie via a ‘top‐down’ approach) following consultation with 18 clinical experts through five in‐person workshops and subsequently validated through follow‐up discussions.

All other disease stages were estimated via a ‘bottom‐up’ approach. This involved estimating the number of times a NASH patient used each health service and health product for the purpose of managing their NASH, and the unit cost of these services and products. These estimates were produced for newly diagnosed and in‐monitoring patients, within each disease stage and for each of the five European countries. Health system utilisation data were extracted from two cost utility studies, containing information on NASH patient management and resource utilisation in the UK.^17^, ^20^ Tanajewski et al and Crossan et al estimated health system utilisation using evidence from scientific literature and clinical practice guidelines from the National Institute for Health and Care Excellence (NICE), European Association for the Study of the Liver (EASL) and American Association for the Study of Liver Disease (AASLD).^17^, ^20^ These sources were supplemented with expert opinion sought from a panel of clinical experts in each of the five countries studied via a series of consultations leveraging principles of expert elicitation to accurately reflect how NASH patients are diagnosed and monitored in each country.^17^

Please refer to File S1 for detailed information regarding the health system utilisations and unit costs derived for this study, including sources.

#### 2.2.2 Productivity costs of diagnosed NASH

Productivity costs were estimated via a human capital approach and include reduced workforce participation, lost productive time caused by absenteeism and presenteeism, forgone income because of premature mortality and search, hiring and training costs.^55^, ^56^, ^57^, ^58^, ^59^, ^60^ This approach involved estimating the number of hours of productivity that are lost owing to NASH. This was then converted into a monetary value by multiplying the number of hours by average weekly earnings adjusted for age, gender and general population employment rates.^55^ It is recognised that the attribution of productivity costs to NASH in comparison to the associated comorbidity load of the patient cohort is complex, and literature sources, which controlled for comorbidities were targeted in the search strategy.

The impact of premature death on workforce participation is captured by forgone future income and does not impact absenteeism, presenteeism or reduced workforce participation. Productivity costs incurred through reduced workforce participation were estimated by applying reduced workforce participation attributable to NASH to the respective five European countries’ general population employment rates and average weekly earnings by age and gender. In lieu of country‐specific estimates, reduced workforce participation attributable to ESLD comprising DCC to liver transplant was obtained from a cross‐sectional analysis of 230 406 adult US Medical Expenditure Panel Survey (MEPS) participants with chronic liver disease including NAFLD and NASH.^56^

Similarly, costs incurred through absenteeism and/or presenteeism were estimated by multiplying the average number of weeks of productive time lost by average weekly earnings. Absenteeism and presenteeism estimates for disease stages F0 to liver transplant were informed by a retrospective analysis of the Adelphi NASH Disease Specific Programme^™^ (DSP), a large, multinational, point‐in‐time survey of physicians and their patients in a real‐world clinical settings conducted from January through June 2018 in the five European countries. A total of 296 physicians (139 hepatologists and 157 gastroenterologists) provided data for 2060 NASH patients and the methodology has been described in detail and validated previously.^58^, ^59^ Each physician completed record forms for 7 patients presenting to them for routine care, capturing clinical details including tests conducted and associated values. Patients were eligible if they were over 18 years old, had a physician‐confirmed NASH diagnosis (via liver biopsy or a non‐invasive test) and were not participating in a clinical trial at the time of the survey. Patients were also invited to complete a voluntary self‐reported questionnaire including the Work Productivity and Activity Impairment (WPAI) validated measure.^60^

Of the 2060 patients included in the NASH DSP, 724 patients qualified for analysis with physician‐reported clinical test values for fibrotic assessment and a corresponding patient‐reported questionnaire capturing WPAI responses. Retrospective WPAI analysis from the NASH DSP included the following: the dataset included patients with disease stages F0‐F4 CC, hence estimates from disease stage F4 CC were extrapolated to include DCC, HCC and liver transplant disease stages; patients with type 2 diabetes were removed; patients were classified by F‐stage severity (F0‐1, F2, F3, F4) via retrospective clinical assessment based on clinical test values (early fibrosis, indeterminate, advanced fibrosis) to ensure correct severity classification.

The NASH DSP obtained ethics approval from the Freiburg Ethics Commission International (FEKI; approval no. 017/1931) for five European countries in 2017.^59^ All patients provided written informed consent for use of their aggregated data.^59^

#### 2.2.3 Other economic costs of diagnosed NASH

Other economic costs estimated include formal and informal care costs, deadweight losses and other financial costs such as funeral costs brought forward caused by premature mortality.^61^, ^62^, ^63^, ^64^, ^65^, ^66^, ^67^, ^68^, ^69^, ^70^, ^71^, ^72^, ^73^, ^74^, ^75^, ^76^, ^77^, ^78^

The average cost of formal care received by NASH patients with ESLD was based on a retrospective cost‐of‐illness study of patients with chronic liver disease conducted over 1 year in Italy, adjusted to 2018 euros.^63^ The opportunity cost method was used to estimate the cost of informal care. This method measures the value of the alternative use of time spent caring, which is typically valued by productivity losses (or value of leisure time) associated with caring. It assumes that time spent providing informal care could be alternatively used within the paid workforce or in leisure activities. The proportion of NASH patients with ESLD who received informal care was obtained from Scalone et al.^63^ The average time spent on informal care was estimated using country‐specific sources.^61^, ^62^ Informal care requirements were assumed to apply evenly across age and gender, varying only by disease stage. The age and gender adjusted average weekly earnings of primary carers was obtained from government sources.^61^, ^62^

Deadweight losses were estimated from inefficiencies associated with forgone taxation revenue and transfer payments.^64^, ^65^ Transfer payments estimated include government expenditure on healthcare and welfare. The number and value of claims each year attributable to NASH was calculated using government sources, adjusted for the proportion of total liver disease in the respective five European countries, which is owing to NASH.^66^, ^67^, ^68^, ^69^, ^70^, ^71^, ^72^, ^73^ Funeral costs brought forward caused by premature mortality were sourced from country‐specific sources and adjusted to 2018.^74^, ^75^, ^76^, ^77^, ^78^

### 2.3 Disease burden and wellbeing costs of diagnosed NASH

Wellbeing costs were estimated using the World Health Organization (WHO) burden of disease methodology and converted into euros using an estimate of the value of a statistical life year (VSLY).^79^, ^80^, ^81^ This is a non‐financial approach, where pain, suffering and premature mortality are measured in terms of disability‐adjusted life years (DALYs).

DALYs are composed of premature mortality (years of life lost owing to premature death—YLL) and morbidity (years of healthy life lost owing to disability—YLD) components. DALYs are calculated by assigning disability weights to various health states, where zero represents a year of perfect health and one represents death. Disability weights for DCC and HCC were sourced from the Institute for Health Metrics and Evaluation (IHME) Global Burden of Disease Study.^79^ Several disability weights were available for HCC depending on the state of the disease (diagnosis compared with terminal disease). Disability weights for HCC were weighted according to the proportion of time spent in each state. DALYs are discounted at a rate of 3% consistent with WHO methodology.^80^

The burden of disease as measured in DALYs was converted into euros using an estimate of the VSLY. The VSLY is an estimate of the value society places on an anonymous life. A per person VSLY for each of the five European countries was obtained from government published sources, or literature, and inflated where required.^81^, ^82^, ^83^

## 3 RESULTS

### 3.1 Diagnosed NASH population

Based on the published prevalence of NASH in adults in the five European countries studied, experts estimates on the subgroup of diagnosed patients were as follows: 5.6%‐5.7% (France), 24.2% (Germany), 3.7%‐3.8% (Italy), 1.9% (Spain) and 20.3% (UK) (Table 2). Likewise, estimates of the proportion of the diagnosed population with advanced fibrosis was as follows: 22.7% (France), 65.8% (Germany), 11.7% (Italy), 1.3% (Spain) and 73.8% (UK) (Tables 1 and 2).

**TABLE 1:** Diagnosed population results (derived probability of diagnosis scenario), people (% of total population)

Disease stage	France	Germany	Italy	Spain	UK	Total
Total general adult (18+ years of age) population	52 405 723	69 833 051	50 891 084	38 144 350	52 403 344	263 677 552
Higher‐prevalence scenario						
F0	9921 (0.02%)	67 925 (0.10%)	2820 (0.01%)	3781 (0.01%)	9841 (0.02%)	94 287 (0.04%)
F1	14 475 (0.03%)	157 345 (0.23%)	3761 (0.01%)	5014 (0.01%)	15 417 (0.03%)	196 010 (0.07%)
F2	7481 (0.01%)	96 470 (0.14%)	2179 (<0.01%)	2877 (0.01%)	71 714 (0.14%)	180 721 (0.07%)
F3	29 275 (0.06%)	200 507 (0.29%)	30 768 (0.06%)	2055 (0.01%)	157 635 (0.30%)	420 239 (0.16%)
F4 CC	34 695 (0.07%)	146 733 (0.21%)	23 929 (0.05%)	2046 (0.01%)	160 358 (0.31%)	367 761 (0.14%)
DCC	9584 (0.02%)	21 334 (0.03%)	18 555 (0.04%)	11 301 (0.03%)	18 652 (0.04%)	79 426 (0.03%)
HCC	644 (<0.01%)	1443 (<0.01%)	1394 (<0.01%)	894 (<0.01%)	1402 (<0.01%)	5777 (<0.01%)
Liver transplant	365 (<0.01%)	206 (<0.01%)	353 (<0.01%)	336 (<0.01%)	200 (<0.01%)	1460 (<0.01%)
Lower‐prevalence scenario						
F0	5953 (0.01%)	23 691 (0.03%)	1070 (<0.01%)	2142 (0.01%)	5304 (0.01%)	38 161 (0.01%)
F1	8685 (0.02%)	54 879 (0.08%)	1427 (<0.01%)	2841 (0.01%)	8310 (0.02%)	76 142 (0.03%)
F2	4489 (0.01%)	33 647 (0.05%)	827 (<0.01%)	1630 (<0.01%)	38 655 (0.07%)	79 248 (0.03%)
F3	17 565 (0.03%)	69 933 (0.10%)	11 678 (0.02%)	1164 (<0.01%)	84 969 (0.16%)	185 309 (0.07%)
F4 CC	20 817 (0.04%)	51 178 (0.07%)	9082 (0.02%)	1159 (<0.01%)	86 437 (0.16%)	168 673 (0.06%)
DCC	5750 (0.01%)	7441 (0.01%)	7043 (0.01%)	6404 (0.02%)	10 054 (0.02%)	36 692 (0.01%)
HCC	387 (<0.01%)	503 (<0.01%)	529 (<0.01%)	507 (<0.01%)	756 (<0.01%)	2681 (<0.01%)
Liver transplant	365 (<0.01%)	206 (<0.01%)	353 (<0.01%)	336 (<0.01%)	200 (<0.01%)	1460 (<0.01%)

Note

Numbers may not sum because of rounding.

Abbreviations: DCC, decompensated cirrhosis; F0, fibrosis stage 0; F1, fibrosis stage 1; F2, fibrosis stage 2; F3, fibrosis stage 3; F4 CC, fibrosis stage 4 compensated cirrhosis; HCC, hepatocellular carcinoma; UK, United Kingdom.

**TABLE 2:** Diagnosed population results (millions) (derived probability of diagnosis scenario)

	France	Germany	Italy	Spain	UK	Total
Higher‐prevalence scenario (% adult population)	3.6	4.1	4.4	3.9	4.1	
NASH diagnosed as % of overall prevalence	5.6	24.2	3.7	1.9	20.3	12.7
F3‐F4 CC diagnosed as % of overall F3‐F4 CC prevalence	22.7	65.8	11.7	1.3	73.8	39.1
NASH diagnosed	0.1	0.7	0.1	0.03	0.4	1.3
F3‐F4 CC diagnosed	0.1	0.3	0.1	0.004	0.3	0.8
Lower‐prevalence scenario (n, % adult population)	2.2	1.4	1.7	2.2	2.2	
NASH diagnosed as % of overall prevalence	5.7	24.2	3.8	1.9	20.3	11.8
F3‐F4 CC diagnosed as % of overall F3‐F4 CC prevalence	22.7	65.8	11.7	1.3	73.8	37.8
NASH diagnosed	0.1	0.2	0.03	0.02	0.2	0.6
F3‐F4 CC diagnosed	0.04	0.1	0.02	0.002	0.2	0.4

Note

Numbers may not sum because of rounding.

Abbreviations: F3, fibrosis stage 3; F4 CC, fibrosis stage 4 compensated cirrhosis; NASH, non‐alcoholic steatohepatitis; UK, United Kingdom.

### 3.2 Economic costs of diagnosed NASH

The following results are reported using the derived probabilities of diagnosis considering a lower‐ and higher‐prevalence scenarios respectively. Results pertaining to the UK were converted from pounds to euros for comparison.^82^ Total economic costs of diagnosed NASH were estimated to range between €1234 and 2037 M (France), €3654 and 10 321 M (Germany), €696 and 1788 M (Italy), €435 and 724 M (Spain) and €2530 and 4676 M (UK). Of these, health system costs were €53‐80 M (France), €210‐561 M (Germany), €82‐183 M (Italy), €53‐69 M (Spain) and €222‐398 M (UK) (Table 3). Average per person health system costs of diagnosed NASH in the five European countries were estimated to range between €699 and 771 (France), €795 and 852 (Germany), €1915 and 2242 (Italy), €1919 and 2568 (Spain) and €890 and 918 (UK) (Table 4). The majority of health system costs were incurred in secondary healthcare, followed by diagnostic tests, primary healthcare and pharmaceuticals (Table 3).

**TABLE 3:** Economic costs results (total, €M) (derived probability of diagnosis scenario)

	France	Germany	Italy	Spain	UK	Total
Higher‐prevalence scenario						
Health system costs	80	561	183	69	398	1291
F3‐CC (% total costs incurred)	31%	15%	13%	3%	56%	28%
ESLD (% total costs incurred)	94%	96%	99%	99%	91%	95%
Primary healthcare	0.05	13	1	0.5	24	39
Secondary healthcare and disease stage	70	472	160	67	249	1018
Diagnostic test	9	73	13	2	99	196
Pharmaceutical	0.5	4	8	0.1	24	37
Medical research	0	0	0	0	2	2
Productivity and other economic costs	1957	9760	1605	655	4278	18 254
F3‐CC (% total costs incurred)	63%	50%	53%	13%	71%	55%
ESLD (% total costs incurred)	77%	56%	92%	71%	80%	65%
Total economic costs	2037	10 321	1788	724	4676	19 546
F3‐CC (% total costs incurred)	61%	48%	45%	11%	69%	53%
ESLD (% total costs incurred)	78%	59%	93%	77%	81%	68%
Lower‐prevalence scenario						
Health system costs	53	210	82	53	222	619
F3‐CC (% total costs incurred)	28%	14%	11%	2%	55%	28%
ESLD (% total costs incurred)	95%	97%	99%	99%	91%	95%
Primary healthcare	0.03	4	0.4	0.3	13	18
Secondary healthcare and disease stage	47	179	73	52	140	491
Diagnostic test	6	26	5	1	54	92
Pharmaceutical	0.3	1	3	0.04	13	17
Medical research	0	0	0	0	2	2
Productivity and other economic costs	1181	3444	614	382	2308	7928
F3‐CC (% total costs incurred)	62%	50%	53%	13%	71%	56%
ESLD (% total costs incurred)	77%	56%	92%	71%	80%	68%
Total economic costs	1234	3654	696	435	2530	8548
F3‐CC (% total costs incurred)	60%	47%	43%	10%	69%	53%
ESLD (% total costs incurred)	79%	59%	93%	78%	81%	70%

Note

Numbers may not sum because of rounding.

Abbreviations: CC, compensated cirrhosis; ESLD, end‐stage liver disease; F3, fibrosis stage 3; UK, United Kingdom.

**TABLE 4:** Economic costs results (per person, €) (derived probability of diagnosis scenario)

	France	Germany	Italy	Spain	UK	Average
Higher‐prevalence scenario						
Health system costs	699	795	1915	1919	890	1244
F0‐F2	147	61	294	88	365	191
F3‐CC	391	235	429	494	704	451
ESLD	912	1411	2079	2793	1036	1646
Productivity and other economic costs	17 093	13 831	16 800	18 134	9560	15 083
F0‐F2	7423	10 403	6476	6092	5565	7192
F3‐CC	10 212	11 016	6606	8104	5974	8382
ESLD	20 825	16 703	17 842	23 886	10 666	17 984
Total economic costs	17 791	14 626	18 715	20 052	10 451	16 327
F0‐F2	7570	10 464	6770	6180	5930	7383
F3‐CC	10 603	11 251	7035	8598	6678	8833
ESLD	21 736	18 114	19 921	26 679	11 701	19 630
Lower‐prevalence scenario^a^						
Health system costs	771	852	2242	2568	918	1470
F0‐F2	147	61	294	88	382	194
F3‐CC	391	235	429	494	705	451
ESLD	1011	1514	2437	3740	1066	1954
Productivity and other economic costs	17 157	13 985	16 832	18 521	9 565	15 212
F0‐F2	7486	10 551	6504	6561	5567	7334
F3‐CC	10 275	11 165	6634	8573	5977	8525
ESLD	20 879	16 859	17 868	24 175	10 671	18 090
Total economic costs	17 928	14 837	19 074	21 089	10 483	16 682
F0‐F2	7633	10 613	6798	6649	5949	7528
F3‐CC	10 666	11 400	7063	9067	6682	8976
ESLD	21 890	18 373	20 305	27 915	11 737	20 044

Abbreviations: CC, compensated cirrhosis; ESLD, end‐stage liver disease; F0, fibrosis stage 0; F2, fibrosis stage 2; F3, fibrosis stage 3; UK, United Kingdom.

^a^ Per‐person costs are slightly higher in the lower prevalence scenario as fixed costs are distributed over a smaller population.

As reported, 58.6% of all NASH patients diagnosed with advanced fibrosis as a result of NASH (F3 to F4 CC) incurred approximately 27.5% of all health system costs (direct costs), 55.2% of productivity and other economic costs (indirect costs) and 52.6% of total economic costs (excluding those associated with death) in 2018 (Table 3). This disease burden further increases in patients with ESLD from NASH (DCC, HCC and liver transplant). It is estimated that ESLD patients represent only 7.4% of all prevalent persons with NASH; however, they constitute 65.0% of all diagnosed NASH patients in the higher case scenario. This patient set was estimated to incur 95.1% of health system costs, 65.3% of productivity and other economic costs and 68.2% of total economic costs (excluding those associated with death) in 2018 (Table 3).

If 100% of the prevalent NASH population was diagnosed in 2018, this cost‐of‐illness study estimated that total economic costs would have totalled €8319‐13 845 M (France), €10 824‐30 878 M (Germany), €5388‐14 150 M (Italy), €4525‐7942 M (Spain) and €7773‐14 404 M (UK). These values may be compared to the costs derived for diagnosed cases only, shown in Table 3. Of these, health system costs were €240‐392 M (France), €265‐719 M (Germany), €345‐876 M (Italy), €186‐305 M (Spain) and €432‐789 M (UK).

The variation in health system costs among the five countries was influenced by differences in the screening, referral, diagnosis and management of NASH patients. For example, NASH patients in the UK received selected pharmaceuticals including pioglitazone, sorafenib, carvedilol, furosemide, spironolactone, rifaximin and lactulose depending on their disease stage and associated complications (Table S12). NASH patients in Germany accessed the same pharmaceuticals with the exception of pioglitazone, which is not used for NASH (Table S13). By contrast, NASH patients in Italy, France and Spain accessed carvedilol (Italy, Spain), pioglitazone (Spain), propranolol (France) and vitamin E (Italy, France and Spain) (Tables S12‐S16).

A marked difference was reported by the experts with regards to the diagnostic test used. While Germany reported imaging measures to screen for HCC in pre‐cirrhotic NASH, other countries relied on AFP and ultrasound only in cirrhotic NASH. Additionally, national choice of biomarker included fibrotest/fibrometer in France, but not in other European countries (Tables S14‐S16).

By comparison, the types of services accessed by NASH patients in a secondary healthcare setting were broadly similar among the five countries costed via a bottom‐up approach. These services included hepatologist consultations and interventions (some of which were surgical) for complications arising from NASH such as HCC, ascites, variceal bleeding and encephalopathy (Tables S12‐S16). However, variation was observed in the use of dieticians and exercise physiologists, as well as in the average utilisation of secondary healthcare services per diagnosed NASH patient.

Similarly, the care received by NASH patients in a primary healthcare setting was almost identical with most patients in the five European countries having seen their general practitioner once following diagnosis and once in monitoring, regardless of disease stage, for the purpose of managing their NASH. Some variation in the utilisation of general practitioner services was observed. Further detail regarding these differences is provided in File S1.

### 3.3 Disease burden and wellbeing costs of diagnosed NASH

In 2018, people with diagnosed NASH in the five European countries were estimated to experience 56 071‐93 451 DALYs in France, 57 932‐166 099 DALYs in Germany, 49 513‐130 453 DALYs in Italy, 54 334‐95 884 DALYs in Spain and 94 094‐174 564 DALYs in the UK. Total wellbeing costs associated with diagnosed NASH in 2018 were estimated to range between €9043 and 15 072 M (France), €9468 and 27 147 M (Germany), €8022 and 21 082 M (Italy), €8760 and 15 458 M (Spain) and €6263 and 11 619 M (UK), and were primarily driven by the high mortality rate of patients with NASH (Table 5).

**TABLE 5:** Disease burden and wellbeing costs results (total) (derived probability of diagnosis scenario)

	France	Germany	Italy	Spain	UK	Total
Higher‐prevalence scenario						
DALYs	93 451	166 099	130 453	95 884	174 564	660 451
Total wellbeing costs (€M)	15 072	27 147	21 082	15 458	11 619	90 379
Lower‐prevalence scenario						
DALYs	56 071	57 932	49 513	54 334	94 094	311 944
Total wellbeing costs (€M)	9043	9468	8002	8760	6263	41 536

Note

Numbers may not sum because of rounding.

Abbreviations: DALYs, disability‐adjusted life years; UK, United Kingdom.

## 4 DISCUSSION

In order to estimate the disease burden and economic impact associated with NASH, a central input is the probability of diagnosis at each stage. NASH in non‐advanced stages is usually asymptomatic and a large proportion of the prevalent population was not diagnosed in 2018.^84^ One major finding of the analysis is a discordant assessment of the experts among the five countries with regards to the number of patients diagnosed with NASH at an advanced stage. These differences are a major aspect behind the numeric difference observed in the cost‐of‐illness analysis. This is mostly impacted by varying patterns of clinical practice within each country with respect to screening, referral, diagnosis and management. Furthermore, the type, frequency and cost of services utilised by patients with NASH differed largely across the five European countries. This means that variation in health system costs estimated can be explained by the fact that different proportions of patients with NASH within each disease stage accessed different combinations of services and products, with differing frequency and unit costs in 2018 and these differences are detailed for the first time in this analysis. Additionally, the analysis supports the need to define patient‐pathways based on best‐practice patterns.^9^

Consultation with local clinical experts revealed that differences in NASH clinical management pathways among the study countries can be attributed to differences in awareness and understanding of NASH among general practitioners and specialists other than hepatologists (eg diabetologists), and the mechanisms by which patients can access specialist medical advice (Tables S11‐S15). For example, in the UK, general practitioners act as ‘gatekeepers’ to specialist medical practitioners such as hepatologists. This means patients are commonly referred to a hepatologist only once a NAFLD diagnosis, or liver‐related concern more generally, is suspected. Once diagnosed, patients are more likely to receive interventions for the purpose of managing NAFLD. Another prominent finding was that patients without advanced fibrosis or cirrhosis are commonly referred back to their general practitioner for management. By comparison, local clinical experts noted that most patients with elevated liver blood test results in Germany would be referred to a hepatologist regardless of other clinical indicators or symptomatology. This is similar to the referral pathway in France whereby the clinical experts consulted noted that the majority of NAFLD patients are referred to specialists by their general practitioner. Also, clinicians in Germany tended to biopsy at an earlier NASH disease stage compared to other European countries. These differences in the pattern of practice are underlying the diagnosed cases in each country.

The local clinical experts noted that primary health physicians in Spain conduct primary screening for NAFLD (Tables S11‐S15). This practice is significantly different in Italy, where significant heterogeneity in clinical practice exists owing to the lack of an established screening process for NASH. This means that, in any given centre, there will be patients who asked their general practitioner for a hepatologist referral, as well as patients who received a hepatologist referral from their general practitioner or other specialist in response to routine test results. This is further complicated by the fact that general practitioners in Italy do not refer patients based on their fibrosis stage, meaning hepatologists may see many F0 and F1 patients and refer them back to their general practitioner for monitoring without making a formal diagnosis. These country‐specific differences underlie, in‐part, the differing probabilities of diagnosis in this study.

Despite these differences, one consistent finding among the included countries was the greater economic burden imposed by ESLD. To this end, this study found that the proportion of diagnosed patients and health system costs were greater in patients with ESLD than in earlier disease stages within the five countries. Importantly, the majority of the undiagnosed population, which makes up approximately 88% of the total prevalence, had early‐stage NASH. As such, patients with early‐stage NASH can be thought of as dormant cases, meaning they incur low costs at present while having the potential to incur significant economic and wellbeing costs in the future after progressing to advanced fibrosis. This is important because the prevalence of NASH is forecasted to increase significantly over time.^6^ Effective management of early‐stage and advanced fibrosis because of NASH patients and preventive measures requires the establishment of a diagnosis. Increasing the proportion of diagnosis for patients with advanced fibrosis caused by NASH, so that they can be counselled for prevention and referred for treatment and active monitoring, will require enhanced awareness and understanding of NASH among general practitioners, specialists other than hepatologists (eg diabetologists) and the general public, in addition to the establishment of best‐practice clinical management pathways within each country.^85^ On the other hand, this is unlikely to be well implemented in clinical practice unless interventions or treatments are available for these patients.

In the literature, three estimates of the economic burden of NAFLD or NASH in the included countries have been published. One such estimate, by Younossi et al, estimated the cost of NAFLD and NASH in France, Germany, Italy, the UK and the US.^2^ In comparison to the current analysis, Younossi et al (2016) estimated the economic burden of NAFLD and NASH using a Markov model of these populations.^2^ Health system costs were estimated for patients with NAFLD; however, productivity and other economic costs were not. Wellbeing costs were estimated by applying the differential in the utility score associated with each disease state and to the willingness‐to‐pay threshold.^2^ Younossi et al found that there were approximately 52 million people with NAFLD across Germany, France, Italy and the UK incurring a total cost of €35B annually. Per‐person direct medical costs for these countries were estimated to range from €354 to 1163.^2^ However, it is important to note that the Younossi et al results are not directly comparable to those presented in this study because of differences in methodology.

A more recent study by Balp et al assessed the comparative burden of NASH in Germany, France, Spain, Italy and the UK relative to a representative sample from the general population and a type 2 diabetes cohort.^86^ In comparison to this study, Balp et al estimated the attributable impact of NASH on health‐related quality of life, WPAI and healthcare resource usage via statistical comparative analysis of the 2016 National Health and Wellness Survey.^86^ Balp et al found that despite NASH patients often having many comorbidities, NASH is independently associated with a significant disease burden and economic impact.^86^ Furthermore, the authors confirm that data on the disease burden and economic impact of NASH are limited at this stage.^86^ Balp et al found that, relative to the general population, patients with NASH experienced worse health‐related quality of life, lower productivity and increased healthcare utilisation.^86^ In addition, Balp et al reported that the prevalence of self‐reported diagnosed NASH equated to 0.29% of the general population across Germany, France, Italy, Spain and the UK.^86^ However, similar to the above, it is important to note that the Balp et al results are not directly comparable to those presented in this study because of differences in methodology.

The third study by O'Hara et al estimated direct medical costs, direct non‐medical costs, indirect costs and patient reported outcomes associated with NASH in Germany, France, Italy, Spain and the UK.^87^ In comparison to this study, O'Hara et al conducted a retrospective, cross‐sectional study that required physician‐recruited patients to provide demographic, clinical and economic information via an online survey.^87^ O'Hara et al found that NASH is associated with reduced quality of life and per patient direct medical, direct non‐medical and indirect costs of €2763, €4917 and €5509, respectively, with the estimated quality of life impacts and costs increasing with disease severity.^87^ Similarly, it is important to note that the O'Hara et al results are not directly comparable to those presented in this study because of differences in methodology.

In addition, a recent systematic review and meta‐analysis by Younossi et al highlights the limited amount of published, peer‐reviewed evidence that is available regarding the prevalence of NASH in the general population.^88^ However, the findings from this study confirm that the prevalence of NASH within the diabetic population is relatively high, with 37.3% of patients with type 2 diabetes estimated to also have comorbid NASH.^88^ This suggests that in addition to incurring significant disease burden and economic costs attributable to NASH, these patients are likely to also incur additional significant socioeconomic costs that are attributable to their diabetes, highlighting the importance of appropriate multidisciplinary management for complex patients with multi‐morbidities.

The current analysis has several limitations related to the unavailability of many of the epidemiological, economic and wellbeing parameters required to model the socioeconomic burden of NASH in the adult population in five European countries in 2018. Owing to these limitations, evidence was derived from studies investigating conditions with comparable aetiology such as cirrhosis (all causes) and countries with comparable demographics and validated via a series of consultations leveraging principles of expert elicitation.^10^, ^11^ This means that in many cases, the inputs underlying this study are uncertain, and changes in these inputs and parameters may have a significant impact upon the total estimate of the costs of NASH in the adult population in the five European countries in 2018. As discussed, NASH is defined by histological changes that can only be assessed by liver biopsy, which is the current ‘reference standard’. Consequently, the prevalence and incidence of NASH has required estimation as a result of a lack of epidemiological literature to use as an input into the modelling process. Furthermore, there are complexities in assigning attribution to NASH directly while excluding the effects of reduced productivity caused by comorbidities such as obesity and diabetes. Productivity inputs used in this study may be an underestimation of the productivity effects attributable to NASH in the five countries. These represent areas for future research. There is a potential for this study to be updated once more data become available. This work also did not include long‐term healthcare costs associated with the growing prevalence of NASH, which has been predicted to increase by 60% by 2030 without intervention.^89^ Similarly, the projected costs associated with the benefits of hypothetical treatment were considered out of scope for this work; however, the reader is referred to a recent paper by Younossi et al on this topic.^90^

Since inputs were validated with leading clinicians and other experts in each country, there was some variation in the inputs and assumptions utilised in the modelling. While we have already described the possible impacts, we do note this is a possible limitation of our methodology. For example, if experts from one country counted cross‐sectional imaging for the evaluation of suspected hepatocellular carcinoma while experts from other countries did not consider this to be related, the estimated economic burden would differ even if the practice is actually similar. Despite this, experts in each country were guided to estimate inputs in a consistent manner (eg by ensuring that each expert considered whether practice truly differed for cross‐sectional imaging), so that the results better reflect true variation in underlying practice rather than a difference in costing approach.

Finally, our study has estimated the burden of diagnosed NASH in each country. This means disease‐modifying interventions in the absence of a confirmed diagnosis have been excluded, although in practice they may occur. For example, a patient with NAFLD and elevated liver enzymes or abnormal liver stiffness may be considered a patient with suspected NASH and some interventions may be offered. Our results should be interpreted noting that these costs have been excluded, although in an ideal world, such a patient would be managed and treated. One of the objectives of this analysis was to highlight the unmet need that arises from undiagnosed NAFLD/NASH in patients and as a consequence the neglect of counselling and appropriate care with regards to their liver disease. On the other hand, this does exclude appropriate care for accompanying comorbidities, and the established management for liver disease is denied to this group.

This study fills an important gap in the literature by providing a comprehensive estimate of the economic and wellbeing cost of NASH in the diagnosed adult population in five European countries in 2018, by disease stage. This is important because while previous studies (described above) have reported on specific costs associated with NASH, they have not included the comprehensive breadth of costs estimated in this study that are important from a societal perspective. Furthermore, many of these studies did not consider the diagnosis rate of NASH, which this study has shown to be an important factor in estimating the total costs attributable to NASH in the present as well as what this low diagnosis rate could mean for costs attributable to NASH in the future. As such, this study highlights that the majority of economic costs are experienced in late disease stages. This means that investments made in preventing progression of patients with advanced fibrosis caused by NASH, to later stages of the disease through optimised screening, referral, diagnosis and management, could bring substantial returns in terms of saved future healthcare costs. To prevent disease progression, pharmacotherapy for advanced stages is eagerly awaited. The results from this cost‐of‐illness study should contribute to educational programmes that increase the awareness of NASH, its associated risks and best‐practice management pathways among general practitioners, specialists other than hepatologists (eg diabetologists) and, ultimately, the general public. The results from this analysis could also provide inputs for screening and treatment (including preventive treatment) reimbursement decisions.

## 5 ETHICS APPROVAL

The NASH DSP obtained ethics approval from the Freiburg Ethics Commission International (FEKI; Approval No. 017/1931) for five European countries in 2017.^59^ All patients provided written informed consent for use of their aggregated data.^59^

## CONFLICT OF INTEREST

Alice Morgan, Sally L Sansom, Sharad Vasudevan and Lynne Pezzullo have received funding from Intercept Pharmaceuticals for consulting services. Jörn Schattenberg reports grants and personal fees from Gilead Sciences; and personal fees from Genfit, Intercept Pharmaceuticals, IQVIA, Novartis, Roche, Bristol‐Myers Squibb and Echosens, outside the submitted work. Jeffrey Lazarus reports personal fees from CEPHEID, GSK and Janssen; grants, personal fees and other from Abbvie and Gilead Sciences; and grants and personal fees from MSD, outside the submitted work. Philip N Newsome reports grants from Novo Nordisk and Boehringer Ingelheim; and other fees from Novo Nordisk, Pfizer, Boehringer Ingelheim, Intercept Pharmaceuticals, Gilead Sciences and Poxel Pharmaceuticals, outside the submitted work. Philip N Newsome is also supported by the National Institute for Health Research (NIHR) Birmingham Biomedical Research Centre at the University Hospitals Birmingham NHS Foundation Trust and the University of Birmingham. The views expressed are those of the author and not necessarily those of the NIHR, the Department of Health and Social Care or the NHS. Lawrence Serfaty reports grants and personal fees from Gilead Sciences; and personal fees from Intercept Pharmaceuticals, AbbVie, Novartis and Pfizer, outside the submitted work. Alessio Aghemo reports grant and research support from AbbVie and Gilead Sciences; and personal fees from MSD, Intercept Pharmaceuticals and AlfaSigma, outside the submitted work. Salvador Augustin reports personal fees from Intercept Pharmaceuticals as part of the submitted work. Salvador Augustin also reports grants and personal fees from Gilead Sciences; and personal fees from Novartis, Pfizer, IQVIA and Allergan, outside the submitted work. Emmanuel Tsochatzis reports personal fees from Intercept Pharmaceuticals, Gilead Sciences and Pfizer, outside the submitted work. Victor de Ledinghen reports grants and non‐financial support from Gilead, AbbVie and Intercept Pharmaceuticals; and personal fees from Gilead, AbbVie, Intercept Pharmaceuticals, Echosens, Supersonic Image, Pfizer, Medac, Indivior, Spimaco, BMS, Bayer and MSD, outside the submitted work. Elisabetta Bugianesi has no conflicts of interest to disclose. Manuel Romero‐Gomez reports grants from AbbVie, Gilead Sciences and Intercept Pharmaceuticals; is a speaker for Gilead Sciences, Intercept Pharmaceuticals, Novo Nordisk, Novartis and Shionogi; is a scientific advisor for Gilead Sciences, Intercept Pharmaceuticals, Novo Nordisk, Prosciento, Kaleido, Boehringer Ingelheim, BMS, Shionogi and Allergan; and is co‐owner of DeMILI, outside the submitted work. Heike Bantel reports personal fees from Intercept Pharmaceuticals, outside the submitted work. Stephen Ryder has attended advisory boards and acted as a paid speaker for Intercept Pharmaceuticals, outside the submitted work. Jerome Boursier has no conflicts of interest to disclose. Javier Crespo reports grant, consultancy and lecture fees from AbbVie, Gilead Sciences, Intercept Pharmaceuticals and MSD. Laurent Castera reports personal fees from AbbVie, Allergan, Echosens, Gilead Sciences, Intercept Pharmaceuticals, Merck, Novo Nordisk, Pfizer and Servier as part of the submitted work. Lefteris Floros has no conflicts of interests to report. Vincenzo Atella has no conflicts of interest to disclose. Jorge Mestre‐Ferrandiz reports personal fees form Intercept Pharmaceuticals as part of the submitted work. Rachel Elliot has no conflicts of interest to disclose. Achim Kautz has no conflicts of interest to disclose. Vlad Ratziu reports other fees from Intercept Pharmaceuticals, outside the submitted work. Victoria Higgins is an employee of Adelphi Real World who have received funding from Intercept Pharmaceuticals for access to the Adelphi NASH DSP data. Aldo Tyrelsinski and Sandrine Cure have received consultancy fees from, and are current employees of, Intercept Pharmaceuticals.

## AUTHORS' CONTRIBUTIONS

The following authors made substantial contributions to the conception and design of the work; the acquisition, analysis and interpretation of data; drafted the reports and manuscript; and provided approval for publication: AM, SLS, SC, SV, LP and AT. The following authors contributed data to the analysis and interpretation of results; critical review of the manuscript drafts; and provided approval for publication: JS, PNN, LS, AA, SA, ET, VdL, EB, MR‐G, HB, SR, JB, JC, LC, LF, VA, JM‐F, RE, AK, JVL, VR and VH. JS and JVL further revised the final drafts. All authors approved the manuscript for publication.

## Supporting information

Supplementary Material

## Data Availability

The datasets supporting the results of this article are available to the public from various government sources. All datasets relied upon have been cited, where appropriate, in the manuscript and included in the reference list. A full report, which outlines all methods and data sources, along with detailed results, is available upon request.
