# Supplementary material for: Disease burden and economic impact of diagnosed non‐alcoholic steatohepatitis in five European countries in 2018: A cost‐of‐illness analysis
Source: Liver Int. 2021 Mar 18;41(6):1227–42. doi: 10.1111/liv.14825 (PMC8252761; doi:10.1111/liv.14825)
Supplement: Supplementary file 1 — Supplementary Material [file LIV-41-1227-s001.docx]

Disease burden and economic impact of diagnosed non-alcoholic steatohepatitis (NASH) in five European countries in 2018: A cost-of-illness analysis

# Additional file 1: Supplementary results

### Expert panel details

Table 1 Expert panel details

| **Country** | **Expert panel** |
| --- | --- |
| France | Hepatologists (6), Health economists (2) |
| Germany | Hepatologists (3), Health economists (1), Patient group advocate (1) |
| Italy | Hepatologists (3), Health economists (2), |
| Spain | Hepatologists (3), Health economists (2), Patient group advocate (1) |
| UK | Hepatologists (3), Health economists (2), Patient group advocates (2) |

### Lower probability of diagnosis scenario

Table 2 Diagnosed population results (lower probability of diagnosis scenario)^†^

| **Disease stage** | **UK** | **Germany** | **Italy** | **France** | **Spain** | **Total** |
| --- | --- | --- | --- | --- | --- | --- |
| **Total general adult (18+ years of age) population** | 52,403,344 | 69,833,051 | 50,891,084 | 52,405,723 | 38,144,350 | 263,677,552 |
| **Higher-prevalence scenario** |  |  |  |  |  |  |
| F0 | 4,920 | 6,792 | 5,641 | 4,960 | 3,781 | 26,095 |
| F1 | 7,708 | 10,490 | 7,521 | 7,237 | 5,014 | 37,970 |
| F2 | 4,346 | 5,847 | 4,359 | 3,741 | 2,877 | 21,169 |
| F3 | 2,706 | 3,439 | 3,077 | 1,952 | 2,055 | 13,229 |
| F4 CC | 3,207 | 3,668 | 3,191 | 1,735 | 2,046 | 13,846 |
| DCC | 17,719 | 20,267 | 17,628 | 9,584 | 11,301 | 76,499 |
| HCC | 1,402 | 1,603 | 1,394 | 758 | 894 | 6,051 |
| Liver transplant | 200 | 206 | 353 | 365 | 336 | 1,460 |
| **Lower-prevalence scenario** |  |  |  |  |  |  |
| F0 | 2,652 | 2,369 | 2,141 | 2,976 | 2,142 | 12,281 |
| F1 | 4,155 | 3,659 | 2,855 | 4,342 | 2,841 | 17,852 |
| F2 | 2,343 | 2,039 | 1,654 | 2,244 | 1,630 | 9,911 |
| F3 | 1,459 | 1,200 | 1,168 | 1,171 | 1,164 | 6,161 |
| F4 CC | 1,729 | 1,279 | 1,211 | 1,041 | 1,159 | 6,419 |
| DCC | 9,551 | 7,069 | 6,690 | 5,750 | 6,404 | 35,465 |
| HCC | 756 | 559 | 529 | 455 | 507 | 2,805 |
| Liver transplant | 200 | 206 | 353 | 365 | 336 | 1,460 |

^†^Numbers may not sum due to rounding. UK, United Kingdom; F0, fibrosis stage zero; F1, fibrosis stage one; F2, fibrosis stage two; F3, fibrosis stage three; F4 CC, fibrosis stage four compensated cirrhosis; DCC, decompensated cirrhosis; HCC, hepatocellular carcinoma.

Table 3 Diagnosed population results (millions) (lower probability of diagnosis scenario)^†^

|  | **UK** | **Germany** | **Italy** | **France** | **Spain** | **Total** |
| --- | --- | --- | --- | --- | --- | --- |
| **Higher-prevalence scenario** |  |  |  |  |  |  |
| NASH diagnosed (% of prevalence) | 2.0 | 1.8 | 1.9 | 1.6 | 1.9 | 1.8 |
| F3-F4 CC diagnosed (% of F3-F4 CC prevalence) | 1.4 | 1.3 | 1.3 | 1.3 | 1.3 | 1.3 |
| NASH diagnosed | 0.04 | 0.05 | 0.04 | 0.03 | 0.03 | 0.2 |
| F3-F4 CC diagnosed | 0.006 | 0.007 | 0.006 | 0.004 | 0.004 | 0.03 |
| **Lower-prevalence scenario** |  |  |  |  |  |  |
| NASH diagnosed (% of prevalence) | 2.0 | 1.8 | 2.0 | 1.6 | 1.9 | 1.9 |
| F3-F4 CC diagnosed (% of F3-F4 CC prevalence) | 1.4 | 1.3 | 1.3 | 1.3 | 1.3 | 1.3 |
| NASH diagnosed | 0.02 | 0.02 | 0.02 | 0.02 | 0.02 | 0.1 |
| F3-F4 CC diagnosed | 0.003 | 0.002 | 0.002 | 0.002 | 0.002 | 0.01 |

^†^Numbers may not sum due to rounding. UK, United Kingdom; NASH, non-alcoholic steatohepatitis; F3, fibrosis stage three; F4 CC, fibrosis stage four compensated cirrhosis.

Table 4 Economic costs results (total, € million) (lower probability of diagnosis scenario)^†^

|  | **UK** | **Germany** | **Italy** | **France** | **Spain** | **Total** |
| --- | --- | --- | --- | --- | --- | --- |
| **Higher-prevalence scenario** |  |  |  |  |  |  |
| Health system costs | 144 | 443 | 159 | 55 | 69 | 869 |
| F3-CC (%) | 3% | 0% | 2% | 3% | 3% | 1% |
| ESLD (F3, F4 CC, DCC, HCC and LT) (%) | 96% | 100% | 97% | 96% | 99% | 98% |
| Primary healthcare | 5 | 2 | 0.4 | 0.003 | 0.5 | 8 |
| Secondary healthcare and disease stage | 111 | 429 | 152 | 54 | 67 | 813 |
| Diagnostic test | 15 | 8 | 4 | 1 | 2 | 30 |
| Pharmaceutical | 11 | 4 | 3 | 0.1 | 0.1 | 18 |
| Medical research | 2 | 0 | 0 | 0 | 0 | 2 |
| Productivity and other economic costs | 2,001 | 3,530 | 1,373 | 1,293 | 655 | 8,850 |
| F3-CC (%) | 10% | 12% | 11% | 12% | 13% | 12% |
| ESLD (F3, F4 CC, DCC, HCC and LT) (%) | 72% | 63% | 71% | 59% | 71% | 66% |
| Total economic costs | 2,144 | 3,973 | 1,531 | 1,347 | 724 | 9,720 |
| F3-CC (%) | 8% | 9% | 8% | 11% | 11% | 9% |
| ESLD (F3, F4 CC, DCC, HCC and LT) (%) | 79% | 72% | 78% | 64% | 77% | 74% |
| **Lower-prevalence scenario** |  |  |  |  |  |  |
| Health system costs | 84 | 168 | 73 | 38 | 53 | 416 |
| F3-CC (%) | 3% | 0% | 1% | 2% | 2% | 2% |
| ESLD (F3, F4 CC, DCC, HCC and LT) (%) | 95% | 100% | 97% | 96% | 99% | 98% |
| Primary healthcare | 2 | 1 | 0.1 | 0.002 | 0.3 | 3 |
| Secondary healthcare and disease stage | 65 | 164 | 70 | 38 | 52 | 389 |
| Diagnostic test | 8 | 3 | 1 | 1 | 1 | 14 |
| Pharmaceutical | 6 | 1 | 1 | 0.04 | 0.04 | 8 |
| Medical research | 2 | 0 | 0 | 0 | 0 | 2 |
| Productivity and other economic costs | 1,080 | 1,271 | 526 | 782 | 382 | 4,041 |
| F3-CC (%) | 10% | 12% | 10% | 12% | 13% | 11% |
| ESLD (F3, F4 CC, DCC, HCC and LT) (%) | 73% | 63% | 71% | 60% | 71% | 66% |
| Total economic costs | 1,165 | 1,439 | 598 | 820 | 435 | 4,457 |
| F3-CC (%) | 8% | 9% | 8% | 10% | 10% | 9% |
| ESLD (F3, F4 CC, DCC, HCC and LT) (%) | 79% | 72% | 79% | 65% | 78% | 74% |

^†^Numbers may not sum due to rounding. UK, United Kingdom; F3, fibrosis stage three; CC, compensated cirrhosis; ESLD, end-stage liver disease; F4 CC, fibrosis stage four compensated cirrhosis; DCC, decompensated cirrhosis; HCC, hepatocellular carcinoma; LT, liver transplant.

Table 5 Economic costs results (per person, €) (lower probability of diagnosis scenario)^†^

|  | **UK** | **Germany** | **Italy** | **France** | **Spain** | **Total** |
| --- | --- | --- | --- | --- | --- | --- |
| **Higher-prevalence scenario** |  |  |  |  |  |  |
| Health system costs | 2,638 | 6,707 | 2,890 | 1,429 | 1,919 | 3,117 |
| Productivity and other economic costs | 36,731 | 53,464 | 24,983 | 33,689 | 18,134 | 33,400 |
| Total economic costs | 39,369 | 60,172 | 27,873 | 35,119 | 20,052 | 36,517 |
| **Lower-prevalence scenario**^‡^ |  |  |  |  |  |  |
| Health system costs | 2,859 | 7,275 | 3,445 | 1,640 | 2,568 | 3,557 |
| Productivity and other economic costs | 36,686 | 54,873 | 24,955 | 33,777 | 18,521 | 33,762 |
| Total economic costs | 39,546 | 62,147 | 28,400 | 35,416 | 21,089 | 37,320 |

^†^Numbers may not sum due to rounding. ^‡^Per person costs are slightly higher in the lower prevalence scenario as fixed costs are distributed over a smaller population. UK, United Kingdom.

Table 6 Disease burden and wellbeing costs results (total) (lower probability of diagnosis scenario)^†^

|  | **UK** | **Germany** | **Italy** | **France** | **Spain** | **Total** |
| --- | --- | --- | --- | --- | --- | --- |
| **Higher-prevalence scenario** |  |  |  |  |  |  |
| DALYs | 174,398 | 165,957 | 130,287 | 93,485 | 95,884 | 660,011 |
| Total wellbeing costs (€ million) | 11,608 | 27,124 | 21,055 | 15,077 | 15,458 | 90,323 |
| **Lower-prevalence scenario** |  |  |  |  |  |  |
| DALYs | 94,005 | 57,883 | 49,450 | 56,091 | 54,334 | 257,429 |
| Total wellbeing costs (€ million) | 6,257 | 9,460 | 7,992 | 9,046 | 8,760 | 41,515 |

^†^Numbers may not sum due to rounding. UK, United Kingdom; DALYs, disability-adjusted life years.

### Higher probability of diagnosis scenario

Table 7 Diagnosed population results (higher probability of diagnosis scenario)^†^

| **Disease stage** | **UK** | **Germany** | **Italy** | **France** | **Spain** | **Total** |
| --- | --- | --- | --- | --- | --- | --- |
| **Total general adult (18+ years of age) population** | 52,403,344 | 69,833,051 | 50,891,084 | 52,405,723 | 38,144,350 | 263,677,552 |
| **Higher-prevalence scenario** |  |  |  |  |  |  |
| F0 | 49,203 | 67,925 | 56,408 | 49,605 | 37,807 | 260,947 |
| F1 | 115,627 | 157,345 | 112,815 | 108,561 | 75,203 | 569,552 |
| F2 | 71,714 | 96,470 | 71,920 | 61,721 | 47,464 | 349,289 |
| F3 | 157,770 | 200,507 | 179,376 | 113,782 | 119,791 | 771,226 |
| F4 CC | 128,286 | 146,733 | 127,623 | 69,389 | 81,820 | 553,852 |
| DCC | 18,652 | 21,334 | 18,555 | 10,089 | 11,896 | 80,525 |
| HCC | 1,261 | 1,443 | 1,255 | 682 | 805 | 5,446 |
| Liver transplant | 200 | 206 | 353 | 365 | 336 | 1,460 |
| **Lower-prevalence scenario** |  |  |  |  |  |  |
| F0 | 26,522 | 23,691 | 21,409 | 29,763 | 21,424 | 122,809 |
| F1 | 62,326 | 54,879 | 42,818 | 65,137 | 42,615 | 267,775 |
| F2 | 38,655 | 33,647 | 27,297 | 37,033 | 26,897 | 163,528 |
| F3 | 85,042 | 69,933 | 68,081 | 68,269 | 67,882 | 359,207 |
| F4 CC | 69,150 | 51,178 | 48,439 | 41,633 | 46,365 | 256,764 |
| DCC | 10,054 | 7,441 | 7,043 | 6,053 | 6,741 | 37,331 |
| HCC | 680 | 503 | 476 | 409 | 456 | 2,525 |
| Liver transplant | 200 | 206 | 353 | 365 | 336 | 1,460 |

^†^Numbers may not sum due to rounding. UK, United Kingdom; F0, fibrosis stage zero; F1, fibrosis stage one; F2, fibrosis stage two; F3, fibrosis stage three; F4 CC, fibrosis stage four compensated cirrhosis; DCC, decompensated cirrhosis; HCC, hepatocellular carcinoma..

Table 8 Diagnosed population results (millions) (higher probability of diagnosis scenario)^†^

|  | **UK** | **Germany** | **Italy** | **France** | **Spain** | **Total** |
| --- | --- | --- | --- | --- | --- | --- |
| **Higher-prevalence scenario** |  |  |  |  |  |  |
| NASH diagnosed (% of prevalence) | 25.3 | 24.2 | 25.4 | 22.0 | 25.2 | 24.4 |
| F3-F4 CC diagnosed (% of F3-F4 CC prevalence) | 66.4 | 65.8 | 65.7 | 65.0 | 65.5 | 65.8 |
| NASH diagnosed | 0.5 | 0.7 | 0.6 | 0.4 | 0.4 | 2.6 |
| F3-F4 CC diagnosed | 0.3 | 0.3 | 0.3 | 0.2 | 0.2 | 1.3 |
| **Lower-prevalence scenario** |  |  |  |  |  |  |
| NASH diagnosed (% of prevalence) | 25.3 | 24.2 | 25.4 | 22.0 | 25.2 | 24.3 |
| F3-F4 CC diagnosed (% of F3-F4 CC prevalence) | 66.4 | 65.8 | 65.7 | 65.0 | 65.5 | 65.7 |
| NASH diagnosed | 0.3 | 0.2 | 0.2 | 0.2 | 0.2 | 1.2 |
| F3-F4 CC diagnosed | 0.2 | 0.1 | 0.1 | 0.1 | 0.1 | 0.6 |

^†^Numbers may not sum due to rounding. UK, United Kingdom; NASH, non-alcoholic steatohepatitis; F3, fibrosis stage three; F4 CC, fibrosis stage four compensated cirrhosis.

Table 9 Economic costs results (total, € million) (higher probability of diagnosis scenario)^†^

|  | **UK** | **Germany** | | **Italy** | | **France** | | **Spain** | | **Total** | |
| --- | --- | --- | --- | --- | --- | --- | --- | --- | --- | --- | --- |
| **Higher-prevalence scenario** |  |  | |  | |  | |  | |  | |
| Health system costs | 390 | 561 | | 360 | | 155 | | 174 | | 1,640 | |
| F3-CC (% total costs incurred) | 50% | 15% | | 37% | | 45% | | 54% | | 35% | |
| ESLD (F3, F4 CC, DCC, HCC and LT)  (% total costs incurred) | 86% | 96% | | 80% | | 79% | | 92% | | 88% | |
| Primary healthcare costs | 34 | 13 | | 8 | | 0.1 | | 9 | | 64 | |
| Secondary healthcare and disease stage costs | 238 | 472 | | 180 | | 121 | | 104 | | 1,115 | |
| Diagnostic test costs | 89 | 73 | | 100 | | 32 | | 60 | | 354 | |
| Pharmaceutical costs | 26 | 4 | | 72 | | 2 | | 2 | | 106 | |
| Medical research costs | 2 | 0 | | 0 | | 0 | | 0 | | 2 | |
| Productivity and other economic costs | 4,840 | 9,760 | | 4,253 | | 4,087 | | 2,561 | | 25,500 | |
| F3-CC (% total costs incurred) | 52% | 50% | | 52% | | 49% | | 59% | | 52% | |
| ESLD (F3, F4 CC, DCC, HCC and LT)  (% total costs incurred) | 60% | 56% | | 60% | | 54% | | 65% | | 58% | |
| Total economic costs | 5,229 | 10,321 | | 4,613 | | 4,242 | | 2,735 | | 27,140 | |
| F3-CC(% total costs incurred) | 52% | 48% | | 51% | | 49% | | 59% | | 50% | |
| ESLD (F3, F4 CC, DCC, HCC and LT)  (% total costs incurred) | 62% | 59% | | 62% | | 55% | | 67% | | 60% | |
| **Lower-prevalence scenario** |  |  | |  | |  | |  | |  | |
| Health system costs | 217 | 210 | | 149 | | 98 | | 112 | | 786 | |
| F3-CC (% total costs incurred) | 49% | 14% | | 34% | | 42% | | 47% | | 35% | |
| ESLD (F3, F4 CC, DCC, HCC and LT)  (% total costs incurred) | 86% | 97% | | 82% | | 80% | | 93% | | 88% | |
| Primary healthcare costs | 18 | 4 | | 3 | | 0.1 | | 5 | | 30 | |
| Secondary healthcare and disease stage costs | 134 | 179 | | 81 | | 77 | | 73 | | 541 | |
| Diagnostic test costs | 48 | 26 | | 38 | | 19 | | 34 | | 165 | |
| Pharmaceutical costs | 14 | 1 | | 27 | | 1 | | 1 | | 44 | |
| Medical research costs | 2 | 0 | | 0 | | 0 | | 0 | | 2 | |
| Productivity and other economic costs | 2,611 | 3,444 | | 1,619 | | 2,459 | | 1,462 | | 11,594 | |
| F3-CC (% total costs incurred) | 52% | 50% | | 52% | | 49% | | 59% | | 52% | |
| ESLD (F3, F4 CC, DCC, HCC and LT)  (% total costs incurred) | 60% | 56% | | 60% | | 54% | | 65% | | 58% | |
| Total economic costs | 2,828 | 3,654 | | 1,768 | | 2,557 | | 1,574 | | 12,380 | |
| F3-CC (% total costs incurred) | 52% | 47% | | 50% | | 49% | | 58% | | 51% | |
| ESLD (F3, F4 CC, DCC, HCC and LT)  (% total costs incurred) | 62% | | 59% | | 62% | | 55% | | 68% | | 61% |

^†^Numbers may not sum due to rounding. UK, United Kingdom; F3, fibrosis stage three; CC, compensated cirrhosis; ESLD, end-stage liver disease; F4 CC, fibrosis stage four compensated cirrhosis; DCC, decompensated cirrhosis; HCC, hepatocellular carcinoma; LT, liver transplant.

Table 10 Economic costs results (per person, €) (higher probability of diagnosis scenario)^†^

|  | **UK** | **Germany** | **Italy** | **France** | **Spain** | **Total** |
| --- | --- | --- | --- | --- | --- | --- |
| **Higher-prevalence scenario** |  |  |  |  |  |  |
| Health system costs | 702 | 795 | 620 | 367 | 455 | 588 |
| Productivity and other economic costs | 8,721 | 13,831 | 7,332 | 9,679 | 6,688 | 9,250 |
| Total economic costs | 9,423 | 14,626 | 7,952 | 10,046 | 7,142 | 9,838 |
| **Lower-prevalence scenario**^‡^ |  |  |  |  |  |  |
| Health system costs | 724 | 852 | 675 | 386 | 517 | 631 |
| Productivity and other economic costs | 8,725 | 13,985 | 7,346 | 9,701 | 6,732 | 9,298 |
| Total economic costs | 9,449 | 14,837 | 8,022 | 10,087 | 7,249 | 9,929 |

^†^Numbers may not sum due to rounding. ^‡^Per person costs are slightly higher in the lower prevalence scenario as fixed costs are distributed over a smaller population. UK, United Kingdom.

Table 11 Disease burden and wellbeing costs results (total) (higher probability of diagnosis scenario)^†^

|  | **UK** | **Germany** | **Italy** | **France** | **Spain** | **Total** |
| --- | --- | --- | --- | --- | --- | --- |
| **Higher-prevalence scenario** |  |  |  |  |  |  |
| DALYs | 174,522 | 166,099 | 130,411 | 93,552 | 95,963 | 660,549 |
| Total wellbeing costs (€ million) | 11,616 | 27,147 | 21,075 | 15,088 | 15,471 | 90,398 |
| **Lower-prevalence scenario** |  |  |  |  |  |  |
| DALYs | 94,072 | 57,932 | 49,497 | 56,131 | 54,379 | 312,012 |
| Total wellbeing costs (€ million) | 6,262 | 9,468 | 7,999 | 9,053 | 8,767 | 41,549 |

^†^Numbers may not sum due to rounding. UK, United Kingdom; DALYs, disability-adjusted life years.

### Health system utilisation and unit cost inputs – United Kingdom (UK)

Table 12 Health system utilisation and unit cost inputs – United Kingdom (UK)

| **Services/products** | **Unit cost (****£)** | **Utilisation per patient** | | | | | | | | **Source** |
| --- | --- | --- | --- | --- | --- | --- | --- | --- | --- | --- |
|  |  | **F0** | **F1** | **F2** | **F3** | **F4 CC** | **DCC** | **HCC** | **LT** |  |
| **Newly diagnosed** |  |  |  |  |  |  |  |  |  |  |
| **Primary healthcare** |  |  |  |  |  |  |  |  |  |  |
| General practitioner (per hour) | 37 | 1 | 1 | 1 | 1 | 1 | 4 |  |  | (1-3, 13) |
| **Secondary healthcare (initial)** |  |  |  |  |  |  |  |  |  |  |
| Hepatologist | 327 | 1 | 1 | 1 | 1 | 1 | 4.5 | 1 |  | (1-3, 11) |
| Dietitian | 46 |  |  |  |  | 1 | 1 |  |  | (1-3, 10) |
| HCC - elective inpatient | 1,810 |  |  |  |  |  |  |  |  | (1-3, 9) |
| HCC - day case | 457 |  |  |  |  |  |  |  |  | (1-3, 11) |
| HCC - chemoembolisation day case | 2,119 |  |  |  |  |  |  | 0.3 |  | (1-3, 11) |
| Ascites - emergency non-elective long stay | 768 |  |  |  |  |  | 0.3 |  |  | (1-3, 11) |
| Ascites - elective inpatient | 1,146 |  |  |  |  |  | 1.0 |  |  | (1-3, 9) |
| Variceal bleed - emergency non-elective long stay | 698 |  |  |  |  |  | 0.1 |  |  | (1-3, 11) |
| Variceal bleed - elective inpatient | 924 |  |  |  |  |  | 0.5 |  |  | (1-3, 9) |
| Encephalopathy - emergency non-elective long stay | 2,315 |  |  |  |  |  | 0.1 |  |  | (1-3, 11) |
| HCC resection - elective inpatient | 5,010 |  |  |  |  |  |  | 0.2 |  | (1-3, 9) |
| Liver transplant | 92,909 |  |  |  |  |  |  |  | 1 | (1-4) |
| Variceal bleed transjugular stent - non-elective short stay | 4,800 |  |  |  |  |  | 0.0 |  |  | (1-3, 11) |
| **Secondary healthcare (follow-up)** |  |  |  |  |  |  |  |  |  |  |
| Hepatologist | 134 | 1 | 1 | 1 | 2 | 2.5 | 4.5 | 4.5 |  | (1-3, 11) |
| HCC-elective inpatient | 1,810 |  |  |  |  |  |  | 1 |  | (1-3, 9) |
| HCC-day case | 457 |  |  |  |  |  |  | 1 |  | (1-3, 11) |
| Nurse (per hour) | 37 |  |  |  |  |  |  | 3 |  | (1-3, 13) |
| HCC resection - elective inpatient (recurrence) | 5,010 |  |  |  |  |  |  | 0.2 |  | (1-3, 9) |
| **Diagnostic tests** |  |  |  |  |  |  |  |  |  |  |
| FIB-4 (liver function test) | 5 | 4 | 4 | 4 | 4 | 4 | 7 |  |  | (1-3) |
| Fibroscan | 48 | 1 | 1 | 1 | 1 | 1 |  |  |  | (1-3, 11) |
| Ultrasound (outpatient) | 48 | 1 | 1 | 1 | 2 | 2 | 2 |  |  | (1-3, 11) |
| Alpha fetoprotein | 5 |  |  |  | 2 | 2 | 2 |  |  | (1-3) |
| Oesophago-gastroduodenoscopy (OGD) | 341 |  |  |  | 0.8 | 0.8 | 1.7 |  |  | (1-3, 11) |
| Liver biopsy | 551 | 0.2 | 0.2 | 0.8 | 0.8 | 0.6 |  |  |  | (1-3, 11) |
| Integrated blood services | 2 | 1 | 1 | 1 | 1 | 1 |  |  |  | (1-3, 9) |
| Immunology | 6 | 1 | 1 | 1 | 1 | 1 |  |  |  | (1-3, 9) |
| **Pharmaceuticals** |  |  |  |  |  |  |  |  |  |  |
| Pioglitazone 30mg per year | 467 | 0.1 | 0.1 | 0.1 | 0.1 | 0.0 |  |  |  | (1-3, 12) |
| Sorafenib | 46,495 |  |  |  |  |  |  | 0.0 |  | (1-3, 12) |
| Carvedilol - variceal bleed | 130 |  |  |  |  | 0.2 | 0.2 |  |  | (1-3, 12) |
| Furosemide - ascites | 27 |  |  |  |  |  | 0.1 |  |  | (1-3, 12) |
| Spironolactone - ascites | 162 |  |  |  |  |  | 0.5 |  |  | (1-3, 12) |
| Rifaximin - encephalopathy | 3,370 |  |  |  |  |  | 0.1 |  |  | (1-3, 12) |
| Lactulose - encephalopathy | 200 |  |  |  |  |  | 0.1 |  |  | (1-3, 12) |
| **In monitoring** |  |  |  |  |  |  |  |  |  |  |
| **Primary healthcare** |  |  |  |  |  |  |  |  |  |  |
| General practitioner (per hour) | 37 | 1 | 1 | 1 | 1 | 1 | 4 |  |  | (1-3, 13) |
| Practice nurse (face-to-face per hour) | 54 | 1 | 1 | 1 |  |  |  |  |  | (1-3, 13) |
| Practice nurse (non-face-to-face per hour) | 11 | 1 | 1 | 1 |  |  |  |  |  | (1-3, 13) |
| **Secondary healthcare (follow-up)** |  |  |  |  |  |  |  |  |  |  |
| Hepatologist | 134 |  |  | 1.4 | 2 | 2 | 4.5 | 5.5 |  | (1-3, 11) |
| Dietitian | 46 |  |  |  |  | 1 | 1 |  |  | (1-3, 10) |
| Nurse (per hour) | 37 |  |  |  | 1 | 1 |  | 3 |  | (1-3, 13) |
| Liver transplant subsequent years | 17,771 |  |  |  |  |  |  |  | 1 | (1-4) |
| HCC - elective inpatient | 1,810 |  |  |  |  |  |  | 1 |  | (1-3, 9) |
| HCC - day case | 457 |  |  |  |  |  |  | 1 |  | (1-3, 11) |
| Ascites - emergency non-elective long stay | 768 |  |  |  |  |  | 1.0 |  |  | (1-3, 11) |
| Ascites - day case | 362 |  |  |  |  |  | 3.1 |  |  | (1-3, 11) |
| Variceal bleed - emergency non-elective long stay | 698 |  |  |  |  |  | 0.1 |  |  | (1-3, 11) |
| Variceal bleed - elective inpatient | 924 |  |  |  |  |  | 0.4 |  |  | (1-3, 9) |
| Encephalopathy - emergency non-elective long stay | 2,315 |  |  |  |  |  | 0.6 |  |  | (1-3, 11) |
| Variceal bleed transjugular stent - non-elective short stay | 4,800 |  |  |  |  |  | 0.0 |  |  | (1-3, 11) |
| HCC resection - elective inpatient (recurrence) | 5,010 |  |  |  |  |  |  | 0.2 |  | (1-3, 9) |
| **Diagnostic tests** |  |  |  |  |  |  |  |  |  |  |
| FIB-4 (liver function test) | 5 | 1 | 1 | 2 | 2 | 2 |  |  |  | (1-3) |
| Fibroscan | 48 |  |  | 1 | 1 | 1 |  |  |  | (1-3, 11) |
| Ultrasound (outpatient) | 48 |  |  |  |  | 2 | 2 |  |  | (1-3, 11) |
| Alpha fetoprotein | 5 |  |  |  |  | 2 | 2 |  |  | (1-3) |
| Oesophago-gastroduodenoscopy (OGD) | 341 |  |  |  |  | 0.5 | 1.6 |  |  | (1-3, 11) |
| **Pharmaceuticals** |  |  |  |  |  |  |  |  |  |  |
| Pioglitazone 30mg per year | 467 | 0.1 | 0.1 | 0.1 | 0.1 | 0.0 |  |  |  | (1-3, 12) |
| Sorafenib | 46,495 |  |  |  |  |  |  | 0.0 |  | (1-3, 12) |
| Carvedilol - variceal bleed | 130 |  |  |  |  | 0.2 | 0.2 |  |  | (1-3, 12) |
| Furosemide - ascites | 27 |  |  |  |  |  | 0.1 |  |  | (1-3, 12) |
| Spironolactone - ascites | 162 |  |  |  |  |  | 0.5 |  |  | (1-3, 12) |
| Rifaximin - encephalopathy | 3,370 |  |  |  |  |  | 0.1 |  |  | (1-3, 12) |
| Lactulose - encephalopathy | 200 |  |  |  |  |  | 0.1 |  |  | (1-3, 12) |

### F0, fibrosis stage zero; F1, fibrosis stage one; F2, fibrosis stage two; F3, fibrosis stage three; F4 CC, fibrosis stage four compensated cirrhosis; DCC, decompensated cirrhosis; HCC, hepatocellular carcinoma; LT, liver transplant; FIB-4, Fibrosis-4.

### Health system utilisation and unit cost inputs – Germany

Table 13 Health system utilisation and unit cost inputs – Germany

| **Services/products** | **Unit cost (€)** | **Utilisation per patient** | | | | | | | | **Source** |
| --- | --- | --- | --- | --- | --- | --- | --- | --- | --- | --- |
|  |  | **F0** | **F1** | **F2** | **F3** | **F4 CC** | **DCC** | **HCC** | **LT** |  |
| **Newly diagnosed** |  |  |  |  |  |  |  |  |  |  |
| **Primary healthcare** |  |  |  |  |  |  |  |  |  |  |
| General practitioner (per hour) | 16.99 | 1 | 1 | 1 | 1 | 1 | 4 |  |  | (1-3, 16) |
| **Secondary healthcare (initial)** |  |  |  |  |  |  |  |  |  |  |
| Hepatologist | 21.43 | 1 | 1 | 1 | 1 | 1 | 1 | 1 |  | (1-3, 16) |
| HCC - elective inpatient | 2,233.07 |  |  |  |  |  |  |  |  | (1-3, 14) |
| HCC - day case | 1,012.51 |  |  |  |  |  |  |  |  | (1-3, 14) |
| HCC - chemoembolisation day case | 3,474.44 |  |  |  |  |  |  | 0.3 |  | (1-3, 14) |
| Ascites - emergency non-elective long stay | 6,886.46 |  |  |  |  |  | 0.3 |  |  | (1-3, 14) |
| Ascites - elective inpatient | 6,886.46 |  |  |  |  |  | 1.0 |  |  | (1-3, 14) |
| Variceal bleed - emergency non-elective long stay | 4,056.98 |  |  |  |  |  | 0.1 |  |  | (1-3, 14) |
| Variceal bleed - elective inpatient | 4,056.98 |  |  |  |  |  | 0.5 |  |  | (1-3, 14) |
| Encephalopathy - emergency non-elective long stay | 6,886.46 |  |  |  |  |  | 0.1 |  |  | (1-3, 14) |
| HCC resection - elective inpatient | 3,911.34 |  |  |  |  |  |  | 0.3 |  | (1-3, 14) |
| Liver transplant | 188,272.74 |  |  |  |  |  |  |  | 1 | (1-3, 5, 15) |
| Variceal bleed transjugular stent - non-elective short stay | 7,517.54 |  |  |  |  |  | 0.0 |  |  | (1-3, 14) |
| **Secondary healthcare (follow-up)** |  |  |  |  |  |  |  |  |  |  |
| Hepatologist | 21.43 | 1 | 1 | 1 | 2 | 2.5 | 3 | 3 |  | (1-3, 16) |
| HCC - elective inpatient | 2,233.07 |  |  |  |  |  |  | 1 |  | (1-3, 14) |
| HCC - day case | 1,012.51 |  |  |  |  |  |  | 1 |  | (1-3, 14) |
| HCC resection - elective inpatient (recurrence) | 3,911.34 |  |  |  |  |  |  | 0.2 |  | (1-3, 14) |
| **Diagnostic tests** |  |  |  |  |  |  |  |  |  |  |
| FIB-4 (liver function test) | 0.75 | 1 | 1 | 1 | 1 | 1 | 1 |  |  | (1-3, 16) |
| Fibrosis Score (NFS) Test | 4.15 | 1 | 1 | 1 | 1 | 1 | 1 |  |  | (1-3, 16) |
| Fibroscan | 50.00 | 1 | 1 | 1 | 1 | 1 |  |  |  | (1-3, 5) |
| Ultrasound (outpatient) | 16.99 | 1 | 1 | 1 | 1 | 2 | 2 |  |  | (1-3, 16) |
| Alpha fetoprotein | 6.40 | 0.8 | 0.8 | 0.8 | 2 | 2 | 2 |  |  | (1-3, 16) |
| Oesophago-gastroduodenoscopy (OGD) | 167.32 |  |  |  |  | 1 | 1.7 |  |  | (1-3, 16) |
| Liver biopsy | 137.45 | 0.2 | 0.2 | 0.2 | 0.8 | 0.3 |  |  |  | (1-3, 16) |
| Integrated blood services | 1.50 | 1 | 1 | 1 | 1 | 1 |  |  |  | (1-3, 16) |
| Immunology | 40.50 |  |  |  | 1 | 1 |  |  |  | (1-3, 16) |
| Computed tomography (CT) | 71.75 |  |  |  |  |  | 0.1 |  |  | (1-3, 16) |
| Magnetic resonance imaging (MRI) | 131.28 |  |  |  |  |  | 0.1 |  |  | (1-3, 16) |
| **Pharmaceuticals** |  |  |  |  |  |  |  |  |  |  |
| Sorafenib | 27,068.40 |  |  |  |  |  |  | 0.0 |  | (1-3, 34) |
| Carvedilol - variceal bleed | 62.05 |  |  |  |  | 0.0 | 0.0 |  |  | (1-3, 34) |
| Furosemide - ascites | 43.80 |  |  |  |  |  | 0.1 |  |  | (1-3, 34) |
| Spironolactone - ascites | 83.95 |  |  |  |  |  | 0.3 |  |  | (1-3, 34) |
| Rifaximin - encephalopathy | 2,361.55 |  |  |  |  |  | 0.0 |  |  | (1-3, 34) |
| Lactulose - encephalopathy | 118.26 |  |  |  |  |  | 0.2 |  |  | (1-3, 34) |
| **In monitoring** |  |  |  |  |  |  |  |  |  |  |
| **Primary healthcare** |  |  |  |  |  |  |  |  |  |  |
| GP (per hour patient contact) | 16.99 | 1 | 1 | 1 | 1 | 1 | 4 |  |  | (1-3, 16) |
| **Secondary healthcare (follow-up)** |  |  |  |  |  |  |  |  |  |  |
| Hepatologist | 21.43 | 1 | 1 | 1.4 | 2 | 2 | 4 | 4 |  | (1-3, 16) |
| Liver transplant subsequent years | 27,229.19 |  |  |  |  |  |  |  | 1 | (1-3, 5, 15) |
| HCC - elective inpatient | 2,233.07 |  |  |  |  |  |  | 1 |  | (1-3, 14) |
| HCC - day case | 1,012.51 |  |  |  |  |  |  | 1 |  | (1-3, 14) |
| Ascites - emergency non-elective long stay | 6,886.46 |  |  |  |  |  | 1.0 |  |  | (1-3, 14) |
| Ascites - day case | 2,954.31 |  |  |  |  |  | 3.1 |  |  | (1-3, 14) |
| Variceal bleed - emergency non-elective long stay | 4,056.98 |  |  |  |  |  | 0.1 |  |  | (1-3, 14) |
| Variceal bleed - elective inpatient | 4,056.98 |  |  |  |  |  | 0.4 |  |  | (1-3, 14) |
| Encephalopathy - emergency non-elective long stay | 6,886.46 |  |  |  |  |  | 0.6 |  |  | (1-3, 14) |
| Variceal bleed transjugular stent - non-elective short stay | 7,517.54 |  |  |  |  |  | 0.0 |  |  | (1-3,14) |
| HCC resection-elective inpatient (recurrence) | 3,911.34 |  |  |  |  |  |  | 0.2 |  | (1-3, 14) |
| **Diagnostic tests** |  |  |  |  |  |  |  |  |  |  |
| FIB-4 (liver function test) | 0.75 | 1 | 1 | 1 | 1 | 1 |  |  |  | (1-3, 16) |
| Fibrosis Score (NFS) Test | 4.15 | 1 | 1 | 1 | 1 | 1 | 1 |  |  | (1-3, 16) |
| Fibroscan | 50.00 | 0.05 | 0.05 | 0.05 | 1 | 1 |  |  |  | (1-3, 5) |
| Ultrasound (outpatient) | 16.99 | 0.05 | 0.05 | 0.05 | 1 | 2 | 2 |  |  | (1-3, 16) |
| Alpha fetoprotein | 6.40 | 0.8 | 0.8 | 0.8 | 1 | 2 | 2 |  |  | (1-3, 16) |
| Oesophago-gastroduodenoscopy (OGD) | 167.32 |  |  |  |  | 1 | 1.6 |  |  | (1-3, 16) |
| Liver biopsy | 137.45 | 0.005 | 0.005 | 0.005 | 0.1 |  |  |  |  | (1-3, 16) |
| Integrated blood services | 1.50 | 1 | 1 | 1 | 1 | 1 |  |  |  | (1-3, 16) |
| **Pharmaceuticals** |  |  |  |  |  |  |  |  |  |  |
| Sorafenib | 27,068.40 |  |  |  |  |  |  | 0.0 |  | (1-3, 34) |
| Carvedilol - variceal bleed | 62.05 |  |  |  |  | 0.0 | 0.0 |  |  | (1-3, 34) |
| Furosemide - ascites | 43.80 |  |  |  |  |  | 0.1 |  |  | (1-3, 34) |
| Spironolactone - ascites | 83.95 |  |  |  |  |  | 0.3 |  |  | (1-3, 34) |
| Rifaximin - encephalopathy | 2,361.55 |  |  |  |  |  | 0.0 |  |  | (1-3, 34) |
| Lactulose - encephalopathy | 118.26 |  |  |  |  |  | 0.173 |  |  | (1-3, 34) |

F0, fibrosis stage zero; F1, fibrosis stage one; F2, fibrosis stage two; F3, fibrosis stage three; F4 CC, fibrosis stage four compensated cirrhosis; DCC, decompensated cirrhosis; HCC, hepatocellular carcinoma; LT, liver transplant; FIB-4, Fibrosis-4; NFS, NAFLD fibrosis score.

### Health system utilisation and unit cost inputs – Italy

Table 14 Health system utilisation and unit cost inputs – Italy

| **Services/products** | **Unit cost (€)** | **Utilisation per patient** | | | | | | | | **Source** |
| --- | --- | --- | --- | --- | --- | --- | --- | --- | --- | --- |
|  |  | **F0** | **F1** | **F2** | **F3** | **F4 CC** | **DCC** | **HCC** | **LT** |  |
| **Newly diagnosed** |  |  |  |  |  |  |  |  |  |  |
| **Primary healthcare** |  |  |  |  |  |  |  |  |  |  |
| General practitioner (per hour) | 15 | 1 | 1 | 1 | 1 | 1 |  |  |  | (1-3, 19, 35) |
| **Secondary healthcare (initial)** |  |  |  |  |  |  |  |  |  |  |
| Hepatologist | 23 | 1 | 1 | 1 | 1 | 1 |  |  |  | (1-3, 18, 22, 35) |
| Dietitian | 23 | 0.5 | 0.5 | 0.5 | 0.5 | 0.5 |  |  |  | (1-3, 18, 22, 35) |
| DCC disease stage | 6,420 |  |  |  |  |  | 1 |  |  | (1-3, 6, 35) |
| HCC disease stage | 12,884 |  |  |  |  |  |  | 1 |  | (1-3, 6, 35) |
| Liver transplant | 91,985 |  |  |  |  |  |  |  | 1 | (1-3, 6, 35) |
| **Secondary healthcare (follow-up)** |  |  |  |  |  |  |  |  |  |  |
| Hepatologist | 18 | 1 | 1 | 1 | 2 | 2 |  |  |  | (1-3, 18, 22, 35) |
| **Diagnostic tests** |  |  |  |  |  |  |  |  |  | (1-3, 18, 35) |
| Fibroscan | 65 | 0.5 | 1 | 1 | 1 | 1 |  |  |  | (1-3, 18, 35) |
| Ultrasound (outpatient) | 73 | 1 | 1 | 1 | 2 | 1.2 |  |  |  | (1-3, 18, 35) |
| Oesophago-gastroduodenoscopy (OGD) | 74 |  |  |  |  | 1 |  |  |  | (1-3, 18, 35) |
| Liver biopsy | 81 | 0.2 | 0.2 | 0.8 | 0.8 | 0.6 |  |  |  | (1-3, 18, 35) |
| Integrated blood services | 18 | 1 | 1 | 1 | 1 | 2 |  |  |  | (1-3, 18, 35) |
| **Pharmaceuticals** |  |  |  |  |  |  |  |  |  |  |
| Carvedilol - variceal bleed | 103 |  |  |  |  | 0.1 |  |  |  | (1-3, 21) |
| Vitamin E | 264 | 0.5 | 0.5 | 0.5 | 0.6 | 0.3 |  |  |  | (1-3, 21) |
| **In monitoring** |  |  |  |  |  |  |  |  |  |  |
| **Primary healthcare** |  |  |  |  |  |  |  |  |  |  |
| GP (per hour patient contact) | 15 | 1 | 1 | 1 | 1 | 1 |  |  |  | (1-3, 19, 35) |
| **Secondary healthcare (follow-up)** |  |  |  |  |  |  |  |  |  |  |
| Hepatologist | 23 | 0.5 | 0.5 | 1 | 2 | 2 |  |  |  | (1-3, 18, 22, 35) |
| Dietitian | 23 | 0.5 | 0.5 | 0.5 | 0.5 | 0.5 |  |  |  | (1-3, 18, 22, 35) |
| DCC disease stage | 6,420 |  |  |  |  |  | 1 |  |  | (1-3, 6, 35) |
| HCC disease stage | 12,884 |  |  |  |  |  |  | 1 |  | (1-3, 6, 35) |
| Liver transplant subsequent years | 17,805 |  |  |  |  |  |  |  | 1 | (1-3, 6, 35) |
| **Diagnostic tests** |  |  |  |  |  |  |  |  |  |  |
| Fibroscan | 65 | 0.5 | 0.5 | 1 | 1 | 0.05 |  |  |  | (1-3, 18, 35) |
| Ultrasound (outpatient) | 73 | 0.5 | 1 | 1 | 2 | 2 |  |  |  | (1-3, 18, 35) |
| Oesophago-gastroduodenoscopy (OGD) | 74 |  |  |  |  | 0.4 |  |  |  | (1-3, 18, 35) |
| Integrated blood services | 18 | 0.5 | 1 | 1 | 1 | 2 |  |  |  | (1-3, 18, 35) |
| **Pharmaceuticals** |  |  |  |  |  |  |  |  |  |  |
| Carvedilol - variceal bleed | 103 |  |  |  |  | 0.1 |  |  |  | (1-3, 21) |
| Vitamin E | 264 | 0.5 | 0.5 | 0.5 | 0.6 | 0.3 |  |  |  | (1-3, 21) |

### F0, fibrosis stage zero; F1, fibrosis stage one; F2, fibrosis stage two; F3, fibrosis stage three; F4 CC, fibrosis stage four compensated cirrhosis; DCC, decompensated cirrhosis; HCC, hepatocellular carcinoma; LT, liver transplant.

### Health system utilisation and unit cost inputs – France

Table 15 Health system utilisation and unit cost inputs – France

| **Services/products** | **Unit cost (€)** | **Utilisation per patient** | | | | | | | | **Source** |
| --- | --- | --- | --- | --- | --- | --- | --- | --- | --- | --- |
|  |  | **F0** | **F1** | **F2** | **F3** | **F4 CC** | **DCC** | **HCC** | **LT** |  |
| **Newly diagnosed** |  |  |  |  |  |  |  |  |  |  |
| **Primary healthcare** |  |  |  |  |  |  |  |  |  |  |
| General practitioner (per hour) | 34.10 |  |  |  |  | 1 |  |  |  | (1-3, 23) |
| **Secondary healthcare (initial)** |  |  |  |  |  |  |  |  |  |  |
| Hepatologist | 149.78 | 1 | 1 | 1 | 1 | 1 |  |  |  | (1-3, 23) |
| Dietitian | 33.38 | 0.2 | 0.2 | 0.2 | 0.2 | 1 |  |  |  | (1-3, 23) |
| Exercise physiologist | 71.88 | 0.01 | 0.01 | 0.01 | 0.01 |  |  |  |  | (1-3, 23) |
| DCC disease stage | 11,884.27 |  |  |  |  |  | 1 |  |  | (1-3, 7, 36) |
| HCC disease stage | 13,178.58 |  |  |  |  |  |  | 1 |  | (1-3, 7, 36) |
| Liver transplant | 68,379.77 |  |  |  |  |  |  |  | 1 | (1-3, 7, 36) |
| **Secondary healthcare (follow-up)** |  |  |  |  |  |  |  |  |  |  |
| Hepatologist | 149.78 | 1 | 1 | 1 | 1 | 2 |  |  |  | (1-3, 23) |
| **Diagnostic tests** |  |  |  |  |  |  |  |  |  |  |
| Fibroscan | 31.29 | 0.95 | 0.95 | 0.95 | 1 | 1 |  |  |  | (1-3, 23-30) |
| Fibrotest/Fibrometer | 47.25 | 0.4 | 0.4 | 0.4 | 1 |  |  |  |  | (1-3, 23-30) |
| Ultrasound (outpatient) | 54.02 | 1 | 1 | 1 | 1 | 2 |  |  |  | (1-3, 23-30) |
| Alpha fetoprotein | 26.46 |  |  |  |  | 1.8 |  |  |  | (1-3, 23-30) |
| Oesophago-gastroduodenoscopy (OGD) | 171.89 |  |  |  |  | 0.8 |  |  |  | (1-3, 23-30) |
| Liver biopsy | 61.61 | 0.1 | 0.1 | 0.2 | 0.4 | 0.7 |  |  |  | (1-3, 23-30) |
| Integrated blood services | 25.65 | 1 | 1 | 1 | 2 | 3 |  |  |  | (1-3, 23-30) |
| **Pharmaceuticals** |  |  |  |  |  |  |  |  |  |  |
| Vitamin E | 51.71 | 0.05 | 0.05 | 0.05 | 0.2 |  |  |  |  | (1-3, 30) |
| Propranolol | 23.21 |  |  |  |  | 0.1 |  |  |  | (1-3, 30) |
| **In monitoring** |  |  |  |  |  |  |  |  |  |  |
| **Primary healthcare** |  |  |  |  |  |  |  |  |  |  |
| General practitioner (per hour) | 34.10 |  |  |  |  |  |  |  |  | (1-3, 23) |
| **Secondary healthcare (follow-up)** |  |  |  |  |  |  |  |  |  |  |
| Hepatologist | 149.78 | 0.5 | 0.5 | 1 | 1.3 | 2 |  |  |  | (1-3, 23) |
| Dietitian | 33.38 | 0.2 | 0.2 | 0.2 | 0.2 |  |  |  |  | (1-3, 23) |
| Exercise physiologist | 71.88 | 0.01 | 0.01 | 0.01 | 0.01 |  |  |  |  | (1-3, 23) |
| DCC disease stage | 17,884.81 |  |  |  |  |  | 1 |  |  | (1-3, 7, 36) |
| HCC disease stage | 13,178.58 |  |  |  |  |  |  | 1 |  | (1-3, 7, 36) |
| Liver transplant subsequent years | 2,929.62 |  |  |  |  |  |  |  | 1 | (1-3, 7, 36) |
| **Diagnostic tests** |  |  |  |  |  |  |  |  |  |  |
| Fibroscan | 31.29 | 0.475 | 0.475 | 0.95 | 1.3 | 1 |  |  |  | (1-3, 23-30) |
| Fibrotest/Fibrometer | 47.25 | 0.2 | 0.2 | 0.4 | 1.3 |  |  |  |  | (1-3, 23-30) |
| Ultrasound (outpatient) | 54.02 | 0.025 | 0.025 | 0.1 | 0.75 | 0.4 |  |  |  | (1-3, 23-30) |
| Alpha fetoprotein | 26.46 |  |  |  |  | 2 |  |  |  | (1-3, 23-30) |
| Oesophago-gastroduodenoscopy (OGD) | 171.89 |  |  |  |  | 0.4 |  |  |  | (1-3, 23-30) |
| Liver biopsy | 61.61 |  |  | 0.03 |  |  |  |  |  | (1-3, 23-30) |
| Integrated blood services | 25.65 | 0.5 | 0.5 | 1 | 1.25 | 2 |  |  |  | (1-3, 23-30) |
| **Pharmaceuticals** |  |  |  |  |  |  |  |  |  |  |
| Vitamin E | 51.71 | 0.05 | 0.05 | 0.05 | 0.2 |  |  |  |  | (1-3, 30) |
| Propranolol | 23.21 |  |  |  |  | 0.1 |  |  |  | (1-3, 30) |

F0, fibrosis stage zero; F1, fibrosis stage one; F2, fibrosis stage two; F3, fibrosis stage three; F4 CC, fibrosis stage four compensated cirrhosis; DCC, decompensated cirrhosis; HCC, hepatocellular carcinoma; LT, liver transplant.

### Health system utilisation and unit cost inputs – Spain

Table 16 Health system utilisation and unit cost inputs – Spain

| **Services/products** | **Unit cost (€)** | **Utilisation per patient** | | | | | | | | **Source** |
| --- | --- | --- | --- | --- | --- | --- | --- | --- | --- | --- |
|  |  | **F0** | **F1** | **F2** | **F3** | **F4 CC** | **DCC** | **HCC** | **LT** |  |
| **Newly diagnosed** |  |  |  |  |  |  |  |  |  |  |
| **Primary healthcare** |  |  |  |  |  |  |  |  |  |  |
| General practitioner (per hour) | 92 | 2 | 2 | 2 | 1 | 1 |  |  |  | (1-3, 31) |
| **Secondary healthcare (initial)** |  |  |  |  |  |  |  |  |  |  |
| Hepatologist | 242 | 1 | 1 | 1 | 1 | 1 |  |  |  | (1-3, 31) |
| DCC disease stage | 2,295 |  |  |  |  |  | 1 |  |  | (1-3, 8) |
| HCC disease stage | 6,721 |  |  |  |  |  |  | 1 |  | (1-3, 8) |
| Liver transplant | 123,268 |  |  |  |  |  |  |  | 1 | (1-3, 8) |
| **Secondary healthcare (follow-up)** |  |  |  |  |  |  |  |  |  |  |
| Hepatologist | 148 | 1 | 1 | 1 | 2 | 1 |  |  |  | (1-3, 31) |
| **Diagnostic tests** |  |  |  |  |  |  |  |  |  | (1-3, 31) |
| Fibroscan | 72 | 0.5 | 0.5 | 0.5 | 1 | 1 |  |  |  | (1-3, 31) |
| Ultrasound (outpatient) | 72 | 1 | 1 | 1 | 2 | 2 |  |  |  | (1-3, 31) |
| Alpha fetoprotein | 5 |  |  |  | 0.5 | 0.5 |  |  |  | (1-3, 31) |
| Oesophago-gastroduodenoscopy (OGD) | 1,322 |  |  |  | 0.1 | 0.8 |  |  |  | (1-3, 31) |
| Liver biopsy | 470 | 0.05 | 0.05 | 0.05 | 0.8 | 0.3 |  |  |  | (1-3, 31) |
| Integrated blood services | 20 | 4 | 4 | 4 | 1 | 1 |  |  |  | (1-3, 31) |
| Immunology (standard) | 20 | 1 | 1 | 1 | 1 | 1 |  |  |  | (1-3, 31) |
| Immunology (comprehensive) | 38 | 1 | 1 | 1 | 1 | 1 |  |  |  | (1-3, 31) |
| **Pharmaceuticals** |  |  |  |  |  |  |  |  |  |  |
| Pioglitazone 30mg per year | 523 | 0.0 | 0.0 | 0.0 | 0.0 |  |  |  |  | (1-3, 33) |
| Vitamin E | 225 | 0.1 | 0.1 | 0.1 | 0.1 |  |  |  |  | (1-3, 33) |
| Carvedilol - variceal bleed | 145 |  |  |  |  | 0.0 |  |  |  | (1-3, 33) |
| **In monitoring** |  |  |  |  |  |  |  |  |  |  |
| **Primary healthcare** |  |  |  |  |  |  |  |  |  |  |
| GP (per hour patient contact) | 92 | 1 | 1 | 1 |  | 1 |  |  |  | (1-3, 31) |
| **Secondary healthcare (follow-up)** |  |  |  |  |  |  |  |  |  |  |
| Hepatologist | 242 |  |  |  | 2 | 2 |  |  |  | (1-3, 31) |
| DCC disease stage | 2,295 |  |  |  |  |  | 1 |  |  | (1-3, 8) |
| HCC disease stage | 6,721 |  |  |  |  |  |  | 1 |  | (1-3, 8) |
| Liver transplant subsequent years | 18,015 |  |  |  |  |  |  |  | 1 | (1-3, 8) |
| **Diagnostic tests** |  |  |  |  |  |  |  |  |  |  |
| Fibroscan | 72 |  |  |  | 0.3 | 1 |  |  |  | (1-3, 31) |
| Ultrasound (outpatient) | 72 |  |  |  | 1 | 2 |  |  |  | (1-3, 31) |
| Oesophago-gastroduodenoscopy (OGD) | 1,322 |  |  |  |  | 0.5 |  |  |  | (1-3, 31) |
| Integrated blood services | 20 |  |  |  |  | 1 |  |  |  | (1-3, 31) |
| **Pharmaceuticals** |  |  |  |  |  |  |  |  |  |  |
| Pioglitazone 30mg per year | 523 | 0.0 | 0.0 | 0.0 | 0.0 |  |  |  |  | (1-3, 33) |
| Vitamin E | 225 | 0.1 | 0.1 | 0.1 | 0.1 |  |  |  |  | (1-3, 33) |
| Carvedilol - variceal bleed | 145 |  |  |  |  | 0.0 |  |  |  | (1-3, 33) |

F0, fibrosis stage zero; F1, fibrosis stage one; F2, fibrosis stage two; F3, fibrosis stage three; F4 CC, fibrosis stage four compensated cirrhosis; DCC, decompensated cirrhosis; HCC, hepatocellular carcinoma; LT, liver transplant.

## References

Tanajewski L, Harris R, Harman DJ, Aithal GP, Card TR, Gkountouras G, Berdunov V, Guha IN, Elliott RA. Economic evaluation of a community-based diagnostic pathway to stratify adults for non-alcoholic fatty liver disease: a Markov model informed by a feasibility study. BMJ open. 2017 Jun 1;7(6):e015659.

(2) Crossan C, Tsochatzis EA, Longworth L, Gurusamy K, Davidson B, Rodríguez-Perálvarez M, Mantzoukis K, O'Brien J, Thalassinos E, Papastergiou V, Burroughs A. Cost-effectiveness of non-invasive methods for assessment and monitoring of liver fibrosis and cirrhosis in patients with chronic liver disease: systematic review and economic evaluation. Health Technol Assess. 2015; 19:1-410.

(1-3) Consultation with clinical experts

(4) Ouwens JP, van Enckevort PJ, TenVergert EM, Bonsel GJ, van der Bij W, Haagsma EB, Rutten FF, Slooff MJ, Koëter GH. The cost effectiveness of lung transplantation compared with that of heart and liver transplantation in the Netherlands. Transplant international. 2003 Feb 1;16(2):123-7.

(5) Siebert U, Sroczynski G, German Hepatitis C Model (GEHMO) Group. Effectiveness and cost-effectiveness of initial combination therapy with interferon/peginterferon plus ribavirin in patients with chronic hepatitis C in Germany: a health technology assessment commissioned by the German Federal Ministry of Health and Social Security. International journal of technology assessment in health care. 2005 Jan;21(1):55-65.

(6) Cortesi PA, Ciaccio A, Rota M, Lim JK, De Salvia S, Okolicsanyi S, Vinci M, Belli LS, Mantovani LG, Strazzabosco M. Management of treatment‐naïve chronic hepatitis C genotype 1 patients: a cost‐effectiveness analysis of treatment options. Journal of viral hepatitis. 2015 Feb;22(2):175-83.

(7) Schwarzinger M, Deuffic-Burban S, Mallet V, Pol S, Pageaux GP, Canva-Delcambre V, Deltenre P, Roudot-Thoraval F, Larrey D, Dhumeaux D, Mathurin P. Lifetime Costs attributable to chronic hepatitis C from the French healthcare perspective. Journal of Hepatology. 2013 Apr 1;58:S21-2.

(8) Buti M, Domínguez-Hernández R, Casado MÁ, Sabater E, Esteban R. Healthcare value of implementing hepatitis C screening in the adult general population in Spain. PloS one. 2018;13(11).

(9) National Health Service (NHS) Improvement. Reference costs. (Internet). London: National Health Service (NHS); c2018 (cited 2018 September 27). Available from: https://improvement.nhs.uk/resources/reference-costs/

(10) National Health Service (NHS) Improvement. National tariff payment system 2016/17. (Internet). London: National Health Service (NHS); c2017 (cited 2018 September 27). Available from: https://improvement.nhs.uk/resources/national-tariff-payment-system-201617/

(11) National Health Service (NHS) Improvement. National tariff payment system 2017/18. London: National Health Service (NHS); c2018 (cited 2018 September 27). Available from: https://improvement.nhs.uk/resources/national-tariff-1719/

(12) Joint Formulary Committee. Volume 73 of British National Formulary Series. 73. London: BMJ Group & Pharmaceutical Press; 2017.

(13) Curtis L, Burns A. Unit costs of health and social care 2017, personal social services research unit, University of Kent, Canterbury, 2017.

(14) Institut für das Entgeltsystem im Krankenhaus (InEK).: Diagnosis Related Groups Catalogue (Fallpauschalen-Katalog) 2017. Institut für das Entgeltsystem im Krankenhaus. Siegburg

(15) Das Statistische Bundesamt (Destatis). Consumer price index for Germany (division six) 2018. (Internet). Wiesbaden: Das Statistische Bundesamt; c2018 (cited 2019 April 3). Available from: https://www.destatis.de/EN/FactsFigures/NationalEconomyEnvironment/Prices/ConsumerPriceIndices/Tables_/ConsumerPricesCategories.html?cms_gtp=151232_list%253D2

(16) Kassenärztliche Bundesvereinigung (KBV). Uniform Rating Scale 2018. Berlin; Kassenärztliche Bundesvereinigung; c2018 (cited 2019 April 3). Available from: https://www.kbv.de/html/online-ebm.php

(17) Gastroenterologische Gemeinschaftspraxis Wiescherstraße. Herzlich willkommen auf der Webseite der Gastro-Praxis-Herne. (Internet). Herne: Gastroenterologische Gemeinschaftspraxis Wiescherstraße; (cited 2019 April 3). Available from: https://www.gastro-praxis-herne.de/

(18) Regione Lombardia. Nomenclatore tariffario di specialistica ambulatoriale in vigore dal 1 luglio 2018. (Internet). Milan: Regione Lombardia; c2018 (cited 2019 April 10). Available from: http://www.regione.lombardia.it/wps/portal/istituzionale/HP/servizi-e-informazioni/enti-e-operatori/sistema-welfare/normativa-e-documenti-welfare

(19) Garattini L, Castelnuovo E, Lanzeni D, Viscarra C, di studio DYSCO DV. Durata e costo delle visite in medicina generale: il progetto DYSCO. Farmeconomia. Health economics and therapeutic pathways. 2003 Jun 15;4(2):109-14.

(20) FASDAC. Nomenclatore tariffario 2017.

(21) AIFA Banca Dati Farmaci. The Drug Database. Rome: AIFA Banca Dati Farmaci; (cited 2019 April 10). Available from: https://farmaci.agenziafarmaco.gov.it/bancadatifarmaci

(22) Regione Lazio. Manuale di applicazione del Catalogo Unico Regionale (CUR) delle prestazioni specialistiche prescrivibili. (Internet). Rome: Regione Lazio; 2017 Mar 15 (cited 2019 April 10). Available from: https://www.salutelazio.it/documents/10182/35003625/Unico+Regionale+%28CUR%29/cc37dd7c-bbca-5ac1-3f5e-ad6636ae8b00?version=1.0

(23) L'Assurance Maladie. Honoraires: Données statistiques sur les honoraires des professionnels de santé (PS) libéraux. (Internet). Paris: L'Assurance Maladie; c2017 (cited 2019 April 5). Available from: https://www.ameli.fr/l-assurance-maladie/statistiques-et-publications/donnees-statistiques/professionnels-de-sante-liberaux/honoraires/honoraires-totaux-et-moyens.php

(24) Ameli.fr pour les assure. Le diagnostic de la stéatose hépatique et de la stéato-hépatite non alcooliques. (Internet). Paris: L'Assurance Maladie; c2019 (cited Year Month Day). Available from: https://www.ameli.fr/assure/sante/themes/steatose-hepatique/diagnostic

(25) Haute Autorité de Santé. ACTES ET PRESTATIONS – AFFECTION DE LONGUE DURÉE: Cirrhoses alcooliques. (Internet). Saint-Denis: Haute Autorité de Santé; c2016 (cited 2019 April 5). Available from: https://www.has-sante.fr/portail/upload/docs/application/pdf/2008-11/lap_ald_6_cirrhoses_final.pdf

(26) L'Assurance Maladie. Fiche d'acte abrégée - CODE: HLHB001. (Internet). Paris: L'Assurance Maladie; (cited 2019 April 5). Available from: https://www.ameli.fr/accueil-de-la-ccam/trouver-un-acte/fiche-abregee.php?code=HLHB001

(27) L'Assurance Maladie. Fiche d'acte abrégée - CODE: HLHB002. (Internet). Paris: L'Assurance Maladie; (cited Year Month Day). Available from: https://www.ameli.fr/accueil-de-la-ccam/trouver-un-acte/fiche-abregee.php?code=HLQM002

(28) L'Assurance Maladie. Table Nationale de codage de Biologie. (Internet). Paris: L'Assurance Maladie; (cited 2019 April 5). Available from: http://www.codage.ext.cnamts.fr/cgi/nabm/cgi-fiche?p_code_nabm=0320&p_date_jo_arrete=%25&p_menu=FICHE&p_site=AMELI

(29) Haute Autoritè De Santé. Guide - Affection de longue durée: Cirrhoses. 2008.

(30) L'Assurance Maladie. Liste des Produits et des Prestations. (Internet). Paris: L'Assurance Maladie; (cited 2019 April 5). Available from : http://www.codage.ext.cnamts.fr/codif/tips/index.php?p_site=AMELI

(31) Gisbert R, Brosa M. Spanish Health Costs and cost-effectiveness ratios Database: eSalud. (Internet) Barcelona: Oblikue Consulting, S.L. Barcelona; c2007 (cited 2019 April 9). Available from: http://www.oblikue.com/bddcostes/

(32) Instituto Nacional de Estadistica (INE). Calculation of the Consumer Price Index percentage changes. (Internet). Madrid: Instituto Nacional de Estadistica; c2019 (cited Year Month Day). Available from: https://www.ine.es/varipc/index.do?L=1

(33) Consejo General de Colegios Oficiales de Farmacéuticos. Bot PLUS 2.0. (Internet). Madrid: Consejo General de Colegios Oficiales de Farmacéuticos; (cited 2019 April 9). Available from: https://botplusweb.portalfarma.com

(34) Lauer-Taxe as of April 1, 2019

(35) Istituto Nazionale di Statistica (Istat). Consumer prices for the whole nation (Nic). (Internet). Rome: Istituto Nazionale di Statistica (Istat); c2019 (cited 2019 April 9). Available from: http://dati.istat.it/Index.aspx?QueryId=23063&lang=en#

(36) L’Insee. Indice annuel des prix à la consommation - Base 2015 - Ensemble des ménages - France - Nomenclature Coicop: 06 – Santé. (Internet). Paris: L’Insee; (cited 2019 April 5). Available from: https://www.insee.fr/fr/statistiques/serie/001764780
